# Quantitative non-invasive cell characterisation and discrimination based on multispectral autofluorescence features

**DOI:** 10.1038/srep23453

**Published:** 2016-03-31

**Authors:** Martin E. Gosnell, Ayad G. Anwer, Saabah B. Mahbub, Sandeep Menon Perinchery, David W. Inglis, Partho P. Adhikary, Jalal A. Jazayeri, Michael A. Cahill, Sonia Saad, Carol A. Pollock, Melanie L. Sutton-McDowall, Jeremy G. Thompson, Ewa M. Goldys

**Affiliations:** 1 Quantitative Pty Ltd ABN 17165684186, Beaumont Hills NSW 2155, Australia.; 2 ARC Centre of Excellence for Nanoscale Biophotonics, Macquarie University, North Ryde 2109, NSW Australia; 3 School of Biomedical Sciences, Charles Sturt University, Wagga Wagga, NSW, 2678, Australia; 4 Kolling Institute of Medical Research, Royal North Shore Hospital/Northern Clinical School, University of Sydney, Pacific Hwy, St Leonards NSW 2065, Australia; 5 Robinson Research Institute, School of Paediatrics and Reproductive Health, The University of Adelaide, Medical School, Frome Road, Adelaide, South Australia, 5005, Australia; 6 Australian Research Council Centre of Excellence for Nanoscale Biophotonics and Institute for Photonics and Advanced Sensing, The University of Adelaide, North Terrace, Adelaide, South Australia, 5005, Australia

## Abstract

Automated and unbiased methods of non-invasive cell monitoring able to deal with complex biological heterogeneity are fundamentally important for biology and medicine. Label-free cell imaging provides information about endogenous autofluorescent metabolites, enzymes and cofactors in cells. However extracting high content information from autofluorescence imaging has been hitherto impossible. Here, we quantitatively characterise cell populations in different tissue types, live or fixed, by using novel image processing and a simple multispectral upgrade of a wide-field fluorescence microscope. Our optimal discrimination approach enables statistical hypothesis testing and intuitive visualisations where previously undetectable differences become clearly apparent. Label-free classifications are validated by the analysis of Classification Determinant (CD) antigen expression. The versatility of our method is illustrated by detecting genetic mutations in cancer, non-invasive monitoring of CD90 expression, label-free tracking of stem cell differentiation, identifying stem cell subpopulations with varying functional characteristics, tissue diagnostics in diabetes, and assessing the condition of preimplantation embryos.

The understanding of heterogeneity of cell populations and the impact of these natural variations in understanding disease, drug response, and optimising therapies is a rapidly evolving research field with great potential to impact our lives^1^,^2^,^3^. The existence of subpopulations with unique biological behaviours has been reported across many cell types based on a variety of characterisation approaches^4^,^5^,^6^,^7^. The discovery of such subpopulations inspired research into identifying disease-associated cells in fields as diverse as cancer^4^, injury^8^, and inflammation^9^.

Feature-based high-content analysis of cellular phenotypes is increasingly recognised as a core methodology for the understanding of cellular heterogeneity^5^,^6^,^7^,^10^. However, high-dimensional feature sets thus generated require specialised analysis methods which have so far lagged behind our ability to collect high content image data. Thus, despite the availability of packages for high-dimensional image-based cell analysis supported by trained classifiers such as CellProfiler Analyst, Enhanced Cell Classifier and similar^11^,^12^, a widespread adoption of high content imaging technologies is still limited^13^. The methods described here build on and extend earlier approaches^5^,^6^,^10^ by introducing new methodologies to identify the most informative feature sets^3^,^6^,^7^,^10^,^13^ and applying them to non-invasively obtained and previously unexplored spectral autofluorescence (AF) cellular features.

Label-free non-invasive cell characterisation can be carried out by several imaging modalities, including Raman spectroscopy and Coherent Anti-Stokes Raman spectroscopy (CARS)^14^,^15^, Fourier transform infrared spectroscopy (FTIR)^14^, two-photon fluorescence^16^ or fluorescence-lifetime imaging microscopy (FLIM)^17^. These capital-intensive techniques are powerful but require expert users. In contrast, spectral analysis of cell autofluorescence can be immediately and broadly applied as it only uses common and inexpensive wide-field fluorescence microscopy where endogenous fluorophore signals excited by widely available light sources provide subtle biochemical signatures of cell constituents.

Single photon-excited AF spectra of cells are broad compared with Raman, CARS and FTIR spectra and they are widely regarded as uninformative. However they carry highly relevant biological information, in particular key signatures of cellular metabolism. Endogenous cell fluorophores include but are not limited to nicotinamide adenine dinucleotide (NADH), NADH phosphate (NADPH), flavin adenine dinucleotide (FAD) and flavin mononucleotide (FMD), retinoids including N-retinylidene-N-retinylethanolamine (A2E), cytochrome C, and proteins including abundant species like collagen and elastin. Some of these fluorophores including NADH and flavins bind to cellular proteins which subtly alters their fluorescence spectra. Thus monitoring AF signatures and their cellular distribution provide insights into cellular processes^16^,^18^,^19^.

In this work we show, for the first time, how to non-invasively extract rich, biologically relevant and quantitative information from AF of cells and tissues. AF is first carefully documented by multispectral imaging where a spectrum is taken at each pixel in the image. This generates about a million such spectra from cellular areas with varying molecular composition. Individual cells are segmented out and their images processed to generate multiple, mathematically defined cellular features that capture significant aspects of cell spectra and patterns in their images (see Supplementary Table 1 for the list of cell features used in each section of this work). Our features include principal component abundance values, mean channel intensity ratios, various statistical measures of pixel values, outcomes of unsupervised spectral unmixing, details of co-localisation of unmixed component images, and many others. In contrast, previous works concerned with AF used a single feature only^16^,^19^.

Biological significance of many features used in this work has been recognised previously. For example, our ratios of mean cellular intensity at distinct excitation and emission wavelengths extend the concept behind redox fluorometry^16^,^18^ which uses a measure of the ratio of NADH to flavin as a measure of metabolic rate. Other features reflect differences in abundance of endogenous fluorescent compounds which can be separately identified by spectral unmixing^20^. Other more complicated features include the correlation factor of cell images in different spectral channels, reflecting correlations between cell fluorophores. These can be biologically revealing. For example, it is known that mitochondrial NADH and flavins exist in a tightly regulated equilibrium^21^, hence deviation from this equilibrium either locally, by changes to compartmentalisation, or on a whole cell level may be an indicator of defects in respiratory chain function, or processes associated with mitochondrial biogenesis^22^. We also use similarity or distance measure features to capture the difference between the measured spectra and known fluorophores. This embodies information concerning the relative abundance of fluorescent compounds and purity of compartments. The degree of pixel spectral variation across the cell, the fluorescent image pattern and statistics associated with such features make it possible to partially capture features such as changes in mitochondrial shape or position, or the clustering of lysosomes. These can be highly informative. For example, bright perinuclear rings associated with protein bound mitochondria excited at 330 nm and observed at 450 nm have been related to stem cell competence^23^, while mitochondrial relocation and structural change has been correlated with the levels of reactive oxygen species (ROS) and mitophagy associated with fission and fusion dynamics^24^.

Multidimensional datasets comprising multiple features calculated for each individual cell are analysed here using the mathematical language of vector spaces. Individual features are assigned basis vectors spanning a vector space *V*. The (multiple) feature values for each cell are represented by vectors in *V*, hence cell populations under investigation are represented by vector clusters. We use mathematical operators on the space *V*, in particular Principal Component Analysis (PCA), which mathematically is a data-dependent rotation operator. We also carry out specific data-dependent projections, including a projection of *V* on the first two or three PCA directions, and those defined in the Linear Discriminant Analysis^25^ (LDA) and the Targeted Projection Pursuit^26^ (TPP). These data-dependent mathematical operators project cellular vector clusters on variously optimised directions called canonical variables. The canonical variables satisfy user criteria such as maximising the spread of data points (PCA), ensuring best separation of selected cell groups (LDA) or maximising specific indices of interest (TPP).

Our results demonstrate that simultaneous extraction of tens of quantitative cellular features derived from multispectral AF images of cells and tissues provides a powerful tool to non-invasively address a wide range of biomedical problems such as the existence of biochemical differences between cell populations, detecting certain genetic mutations, label-free quantification and real-time monitoring of cell surface biomarkers, label-free assessment of stem cell differentiation, identifying stem cell subpopulations with varying functional characteristics, tissue diagnostics in metabolic diseases, and quality assessment of preimplantation embryos. Although the mathematical methods presented here have been applied to spectral features only, they can be extended to an arbitrary set of cell features obtained from various microscopy imaging modalities, or other parameters obtained by alternative methodologies, such as clinical data.They also enable a comprehensive analysis of biological problems in a wider analytical context and they can help identify the most predictive parameters in an automated manner. Unrelated data sets (for the same individual cells, or cell groups or patients) obtained by this and other analytical methods can be merged and analysed together, providing a holistic view of the problem under investigation.

## Materials and Methods

### Preparing cells and tissue for spectral imaging

We imaged cultured human cell lines, fixed *in-vitro*-produced cattle preimplantation embryos and freshly excised frozen tissues from diabetic mice and controls prepared as described in Supplementary Note 1. All live cell experiments were conducted using triplicate cultures of all cell types using 35 mm plastic culture dishes with 18 mm well and # 1.5 cover slip bottoms (Cell E&G, USA, Cat number GDB0004-200). These have external 200 μm grids laser etched to assist with cellular relocation for correlative experiments. Each dish is seeded with one ml of trypsinised (5000 cells/cm^2^). Cells in all groups of dishes are incubated at 37 °C, 5% CO_2_ and 90% humidity and measured at ~37 °C in otherwise ambient conditions. Unless otherwise noted, all experiments were carried out at the same cell density, in triplicates. All images have been analysed without subjective selection.

### Spectral fluorescence microscopy

In this work we have employed UV and visible continuous wave epifluorescence microscopy. Excitation wavelengths were supplied by low cost multi-LED light source (Mic-LED Light Source) from Prizmatix Ltd., Givat-Shmuel, Israel. This light source was optical fibre-coupled to the Olympus IX71 microscope. Spectral AF images of biological specimens were obtained at a number of excitation wavelength ranges between 334 and 495 nm, each 10 nm wide. The emission was detected in the range 450 nm-700 nm (see Supplementary Note 2 for details of spectral channels).

An iXon 885 Electron Multiplying Charged Coupled Device (EMCCD) from Andor Technology plc. (Belfast UK) was used to capture images typically using an EM gain sufficient to lift the low light autofluorescence signal above the 17 electrons per pixel per second readout noise but with minimal contribution from clock induced charge. Gain linearity is ensured by using the Andor Real Gain^TM^ technology. The camera is operated below −70 °C such that thermal noise is negligible. Quantum efficiency of the sensor varies from 50–65% across the range of interest from 450–670 nm and the intensity is digitised into ~16 K values.

All images are filtered using customised wavelet filters (see Supplementary Note 3) designed to remove Poisson noise whilst preserving the highly informative phase structure of the image by building on earlier work by Kovesi^27^,^28^. Background signal is subtracted from all images, and was kept minimal through the use of low fluorescence culture dishes (Cell E&G, USA, GDB0004-200). A single wavelength image may take between 1–5 seconds depending on the sample and wavelength but 1–2 minutes is typically required for the entire stack of images for all spectral channels.

### Data analysis methods

We analyse a set of images generated for each field of view comprising spectrally varying fluorescence excitation signals for each pixel in the image. These constitute our primary set of spectral variables, which was further enhanced by including mathematical manipulations of the spectral channel signals, such as ratios of the fluorescence intensities, correlations and other parameters described in detail in Supplementary Note 3 and Supplementary Table 1, again on a pixel-by-pixel basis across the image. In many cases the primary spectral variables and those obtained by their mathematical manipulations reflect distinctive biological properties. Further, the cells wholly contained in each image were segmented and we computed cell features, defined as statistical measures of the spectral variables for each cell, including averages, variances, etc. (see Supplementary Table 1). The multiple features calculated for each cell form multidimensional feature vectors, a dataset mathematically similar to those obtained from flow cytometry. The feature vectors are, generally, correlated in a mathematical sense, requiring decorrelation prior to multivariate analysis^29^.

To analyse these datasets we use linear operators for decorrelating variables (PCA), or maximally separating groups of data based on a distance metric by LDA. PCA is a unitary transformation (a rotation) such that a projection onto the first two or three most informative eigenvectors explains most of the data variability with new uncorrelated variables^30^. The subsequently applied LDA is a projection returning *n-1* discriminating new variables where *n* is the number of distinct groups under investigation, thus the dimensionality of the representation space is investigator-dependent^25^.

We implement our method by using our data covariance matrix in the case of PCA or statistical distance matrix in the case of LDA. A singular value or eigenvalue decomposition of these matrices is performed to obtain eigenvectors which form new basis vectors (referred to as canonical variables^31^). The data are then projected onto these optimised vectors for best visualisation. Therefore canonical variables forming the axes in such figures are linear combinations of our cellular features. Those features which are the combinations of the original spectral channel readings reflect fluorescent colour.

In addition to PCA and LDA we also use the approach of targeted projection pursuit (TPP). The TPP algorithm explores high-dimensional multivariate data to reveal otherwise hidden structures. Mathematically, in TPP, one seeks a new set of basis vectors onto which the data points are projected, so as to satisfy a set of user criteria.

For our hypothesis testing, we carry out LDA with two groups. In this case the data are projected onto a single optimised direction to maximise group separation. This makes it possible to generate histograms and apply statistical tests (such as the Kolmogorov-Smirnov test) that determine if the underlying distributions are different. In this manner the data is presented and analysed to best satisfy interest/relevance, with redundant or uninteresting information being made orthogonal to the plane of projection. Classification performance and more details on hypothesis testing are described in more detail in Supplementary Note 4. In order to identify subpopulations, a simple intuitive algorithm detecting individual data clusters is implemented as described in Supplementary Note 5. The significance threshold in this work was determined at p < 0.05. We performed all image and data analysis by using custom software written in Matlab 2014 (Math works). The Matlab code is available to academic laboratories upon request.

More detailed information on preparation of biological samples, multispectral measurement technique, features used in this work, mathematical methods for identification of subpopulations, classification performance, and additional flow cytometry characterisations is described in Supplementary Material.

## Results and Discussion

### Autofluorescence detects metabolic differences induced by the expression of mutant PGRMC1 protein in pancreatic carcinoma cells

**Figure 1:**
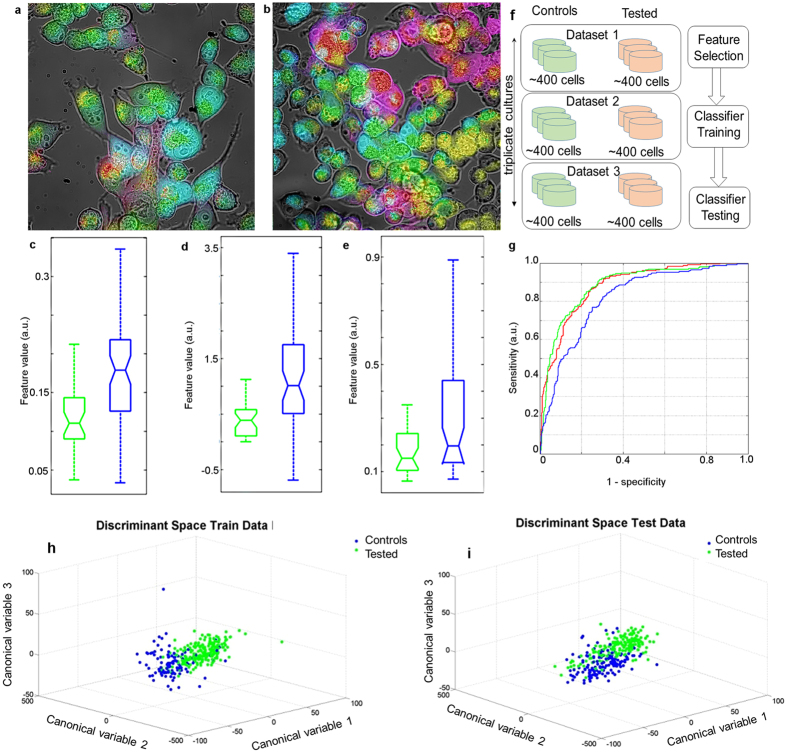
Hyperspectral fluorescence images and data analysis for cultured MiaPaCa-2 pancreatic carcinoma cells. (**a,b**) Images of MiaPaCa-2 pancreatic cancer cells (controls – left and mutated – right, see Supplementary Note 1 for cell culture and mutation details.). The first three PCA variables produced by the PCA transform of AF intensities from eighteen spectral channels in ach pixel were coloured red, green and blue. These images were blended with a corresponding differential interference contrast (DIC) image to show cell membrane and nucleus. The control MiaPaCa-2 pancreatic cancer cells (**a**) appear overall more green/blue whereas the mutated MiaPaCa-2 cells (**b**) appear more yellow/red, clearly highlighting very different spectral AF characteristics between these groups. (**c–e**) Box plot representations of feature distributions obtained from three PCA variables for cells from the dataset shown in (**a**) and (**b**) confirm statistically significant differences. Control MiaPaCa-2 cell feature distributions are shown in green, the mutated MiaPaCa-2 cells in blue. (**c**) The variance of the first principal component; (**d**) The skewness of the 2nd principal component; (**e**) The mean cellular content of the 3rd principal component; (**f**) Schematics of cross-validation. Green dishes: control cells (Mia PaCa-2), pink dishes: tested cells (mutated MiaPaCa-2); (**g**) The ROC for classification using a LOOCV scheme. The three classifiers tested were (**i**) Linear with Area Under Receiver Operating Characteristic (ROC) curve (AUROC) discriminating performance of 0.8806 (p < 0.00001) (red); (ii) Quadratic with AUROC of 0.8824 (p < 0.00001) (green); (iii) naïve Bayes with AUROC of 0.8174 (p < 0.05) (blue)^55^,^56^. (**h,i**) Discrimination of mutant cells (green) from controls (blue). (**h**) Training data projected onto the discriminant space identified by our initial feature selection (see Supplementary Table 1); (**i**) test data projected onto the same discriminant space. Note that discrimination of tested MiaPaCa-2 mutants from MiaPaCa-2 control cells is very well preserved.

As an initial demonstration we have shown that our method is able to clearly distinguish biologically similar cultured cell groups, specifically MiaPaCa-2 pancreatic carcinoma cells (controls) and their genetic mutants expressing a mutated PGRMC1 protein (see Supplementary Note 1for the details of this and subsequent cell cultures and biological interventions). Multispectral imaging was carried out (see Online Methods and Supplementary Note 2) and cellular features were extracted as detailed in Supplementary Note 3. Cell distinction is noticeable in the false colour images of control cells (Fig. 1a) and mutants (Fig. 1b) where red, green and blue colours have been assigned to features with the top 3 PCA scores. Figure 1c–e, quantifies the three most distinctive spectral features in these cell groups derived from PCA components and confirms statistically significant differentiation of mutant and control cells. Note also the correlated difference in cell morphology, with mutant PGRMC1 inducing a rounded phenotype.

To test the robustness of our method to distinguish cell groups, a cross-validation test was employed (Fig. 1f). The test involved partitioning the data into three separate datasets 1, 2 and 3 (different dishes in triplicate cultures, about 400 cell images from each dish). We then performed: (1) feature selection using dataset 1; (2) training a deterministic classifier on dataset 2, and; (3) testing of the classifier on dataset 3 (for details of the procedure see Supplementary Note 4). The classification performance is excellent as shown in Fig. 1g. This figure shows the receiver operator characteristic (ROC) curve for classification using a Leave-One-Out Cross-Validation (LOOCV) scheme where the test and training data are used to train the classifier less one point which is then tested and the procedure repeated across all points. This low-bias scheme is suitable for limited size data^32^. Finally, Fig. 1h,i demonstrates that cellular features suitably chosen by TPP (see Supplementary Table1) and optimised canonical variables derived from them ensure excellent discrimination between control cells and mutants in this experiment. Although no clear biological meaning can be attached to these canonical variables, which are linear combinations of features used in each section where the coefficients are data-dependent, this methodology reveals the existence of large and highly significant differences between the cells. These results confirm that multispectral analysis can be sufficiently nuanced to provide a robust distinction of cell groups in the case of closely similar cells. A more complete characterisation of biological differences between these cells will be published elsewhere.

### A suitable autofluorescence feature can predict CD90 antigen levels in adipose-derived stem cells

**Figure 2:**
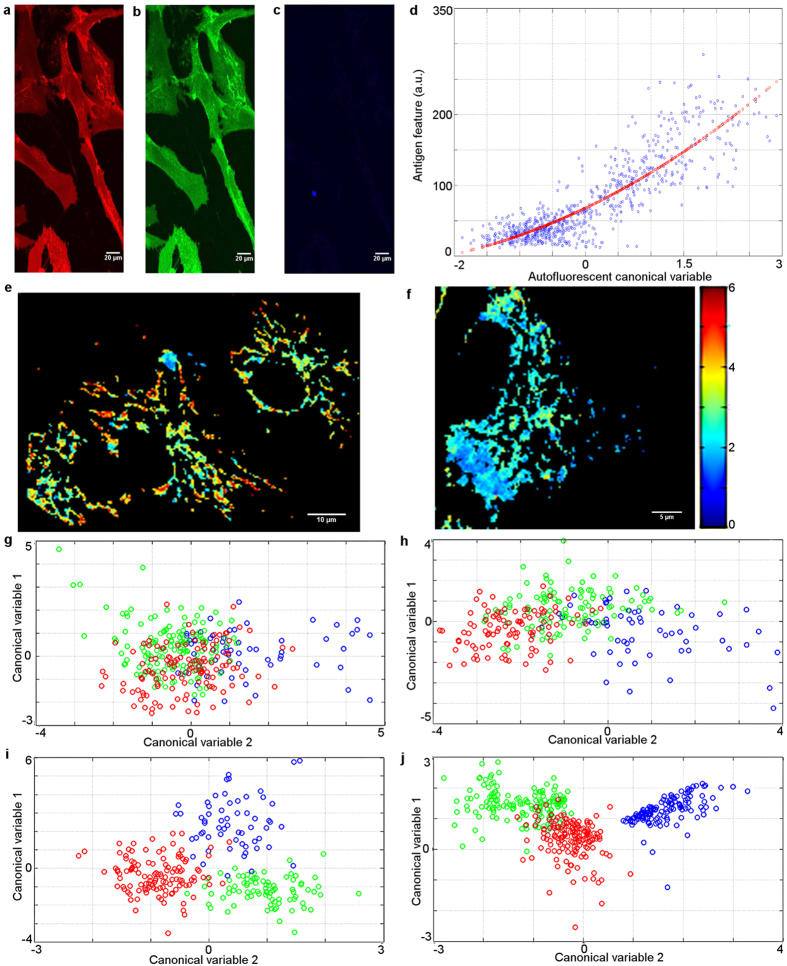
Hyperspectral characterisation of adipose-derived human stem cells before and after osteogenic differentiation. (**a**–**c**) Laser scanning confocal image of ADSCs labelled with CD90 (**a**, red), CD54 (**b**, green), and CD14 (**c**, blue) biomarkers. Stem cells should be positive for CD90 and negative for CD14, while CD54 is useful to identify stem cell subpopulations; (**d**) Label-free detection of CD90. Correlation between the optimised spectral variable and the level of CD90 in cells expressing that biomarker. Data points are average values in each cell. The correlation coefficient is *r* = 0.87; (**e–j**) AF and biomarker characterisation of stem cells before and after osteogenic differentiation.(**e,f**) Spectral band ratio (360 nm to 460 nm excitation), an example AF feature affected by osteogenic differentiation; (**e**) in ADSCs stem cells at 0 h; (**f**) in the same ADSCs 96 hours after differentiation. Panels (**g–i**) show projections of average cell spectral content onto an optimised 2D feature space. Control cells at 3 days + 0 h are shown in blue. (**g**) Control cells 3 days + 24 hrs (green), differentiating ADSCs at 3 days + 24 hrs (that is 4 days after addition of cofactors for differentiation, red). (**h**) Control cells 3 days + 48 hrs later (green), differentiating ADSCs at 3 days + 48 hrs (red). (**i**) Control cells at 3 days + 96 hrs (green), differentiating ADSCs at 3 days + 96 hrs (red). For comparison, panel (**j**) shows the best separation obtained using surface antigen images of CD54, CD90 and CD166. Control cells at 96 h (green), differentiating ADSCs at 96 h (red).

Our method is also capable of providing optimised spectral signatures for biologically significant characterisation of cell surface biomarkers which now can only be done by invasive labelling. Adipose-derived stem cells (ADSCs, Invitrogen) in this and subsequent experiments were obtained from adult adipose tissue by purification of the stromal vascular fraction. The investigated cells were mostly mesenchymal stromal cell (MSC), which was confirmed by labelling with a panel of biomarkers (CD105-PE+, CD73-PE+,CD90-FITC+, CD45-FITC−, CD34-Per5.5−, CD14-Per5.5−,CD19-FITC− and HDLA-DR)^33^ and flow cytometry (Supplementary Note 1 and Supplementary Figures 1–4). The cells were also characterised by confocal imaging using additional antigens, includingCD54-PE, CD133-PE, CD271-FITC and CD166-PerCP (see Fig. 2a–c, and Supplementary Figures 1 and 2). These are known as indicators of differentiation^34^ and for their potential to reveal subpopulations. We measured multispectral AF of these cells and used PCA followed by LDA to find hyperspectral features of cell AF that reflect the level of individual surface biomarkers (see Supplementary Table 1 for the list of applicable features). We found (Fig. 2d) that the level of CD90 is highly correlated (correlation coefficient r = 0.87) with an optimised linear combination of features listed in Supplementary Table 1. This means that AF is able to yield sufficient information to non-invasively identify the level of CD90 on cell membranes with high accuracy.

### Early osteogenic differentiation of adipose-derived stem cells

We now discuss how to non-invasively discriminate undifferentiated ADSCs from the same ADSCs which have been subjected to osteogenic differentiation (see Supplementary Note 1). In this experiment we induced differentiation in tested ADSCs on day 1. The control cells were ADSCs which were allowed to grow for exactly the same time period. Later we took a series of spectral AF images of the following cell groups: control ADSCs at 3 days + 0 h, + 24 h, + 48 h and + 96 h and corresponding differentiated ADSCs at 3days + 0 h, + 24 h, + 48 h and + 96 h after induction of osteogenic differentiation. The spectral data were then used to generate a total of 14 features (see Supplementary Table 1) and we applied the projection and optimisation procedure (PCA and LDA) which best separated the investigated cell groups. These produce two directions which are optimised to the specific dataset under investigation (canonical variables 1 and 2 in Fig. 2). For comparison, in a parallel experiment we labelled cells with CD54, CD90 and CD166, respectively conjugated with three different fluorophores (Fig. 2a–c), and carried out confocal imaging. We used these three fluorophore features to generate an optimal projection that best separates the same cell groups. Figure 2g–i shows projections of average cell spectral AF content vectors onto two canonical variables that best separate the differentiating ADSCs, control ADSCs at the same time point and the ADSCs at t = 0. We see that the non-invasive discrimination of cell groups by AF (Fig. 2g–i) is comparable to the antigen-based discrimination (Fig. 2j), with AF performing better at separating stem cells and the differentiating cells at 96 hours (green and red). Hence, suitably optimised AF features are able to distinguish stem cells from differentiating cells. Earlier work^16^,^35^ has shown that stem cell differentiation is detectable via two-photon AF, but at a reduced separation of cell groups.

We also comment on the shift with time of the cluster of control cells in the feature space (best seen in Fig. 2i). This is related to the fact that that the native fluorophore content in cells evolves as a function of multiple external influences, in particular it is affected by cell confluence which varies over the time of this experiment.

Some of the AF features in this experiment appear informative also at the single cell level. Figure 2e,f show one such example of the band ratio (360 nm/460 nm excitation) which the literature correlates to the NADH/flavin ratio^36^. This feature shows dramatic changes over the course of stem cell differentiation, consistent with metabolically quiescent cells with low level of mitochondrial flavin to a more metabolically active, high flavin state. The stem cell image also shows a higher band ratio in the perinuclear region than on the periphery of the cell, consistent with perinuclear rings of mitochondrial activity. Such bright perinuclear rings associated with protein bound mitochondria exhibiting excitation and emission at wavelengths of 330 nm and 450 nm have been associated with stem cell competence^23^.

### Cell subpopulations within ADSC can be identified in a label-free manner

The ADSCs which are derived from the stromal vascular fraction include mesenchymal stem cells (MSCs) as well as osteoblasts, chondrocytes, and adipocytes^34^.They also contain fibroblasts, a key histological component of adipose tissue^37^ and these lead to gradual loss of osteogenic differentiation potential of ADSCs after passage 5–6^38^. The morphology of fibroblasts *in-vitro* is highly similar to MSCs, making their physical separation very difficult^39^. Fibroblasts have similar genetic and immune-phenotypic surface marker profiles as MSCs, including CD105, CD73, and CD90^40^,^41^,^42^. Here, we employed CD166 and CD54 biomarkers to develop an effective non-invasive identification strategy of selected ADSC subpopulations. CD166 and CD54 antigens have been chosen because stem cells have high expression levels of CD166 while fibroblasts and differentiating stem cells show increased levels of CD54^38^,^43^. The expression of CD54 in MSCs has been reported to increase during osteogenesis by as much as ~200%^34^).

**Figure 3:**
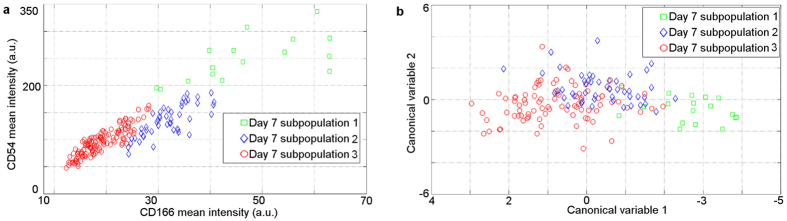
Label-free identification of subpopulations of stem cells. (**a**) Average surface antigen expression levels of CD166 and CD54 for the examined cells. Different symbols indicate automatically identified subpopulations. (**b**)The three subpopulations identified in Fig. 3a, are now shown in a space spanned by two optimised canonical variables. Comparing with Fig. 3a we see similar separation and grouping. Red circles mark cells that have low expression of CD54 and CD166, the blue diamond group show moderate expression CD54 and CD166, and the green squares group show high expression levels of both antigens.

We demonstrate that our method provides a label-free means of detecting rapidly differentiating ADSC subpopulation of ADSCs within a background of fibroblasts and potential other ADSC subpopulations which may differentiate more slowly. To this aim we induced osteogenic differentiation in ADSCs (see Supplementary Note 1). The autofluorescence and subsequent CD166- and CD54-labelled confocal images of cells were taken at various stages of osteogenic differentiation (between day 1 and 7). The multi spectral AF images of living cells were taken first. The same cells were then doubly labelled with CD166 and CD54 and imaged again by confocal microscopy, to determine the level of these biomarkers in the examined cells. Subsequently, the corresponding cells in AF and labelled images were identified and spectral AF features in each of these cells were correlated with the respectiveCD166 and CD54 biomarker level in the same cell. Our unsupervised algorithm (see Supplementary Note 5) applied to labelled images of differentiated and control cells on day 7 automatically found several cell subpopulations (Fig. 3a), which was also confirmed by our flow cytometry analysis of biomarker-labelled cells (Supplementary Figures 1–4). Figure 3a shows that one of these subpopulations (red circles) is characterised by low levels of CD54 and CD166. The second subpopulation (blue diamonds) contains differentiated ADSCs with moderate levels of CD54 and CD166, and the subpopulation of differentiated ADSCs (green squares) exhibits simultaneous high expression of CD166 and CD54. By using PCA and LDA we identified autofluorescence hyperspectral features that best separate these three subpopulations (see Fig. 3b). These spectral features are such that the populations marked with red circles and green squares are entirely non-overlapping. The subpopulations of moderate CD54 and high CD54 cells (blue diamonds and green squares) are also almost separate. These results indicate that different ADSC subpopulations can be distinguished without chemical interference. This new tool can be applied to non-invasively identify and select stem cells with desirable properties for regenerative medicine.

### Distinguishing diabetic from healthy tissue

**Figure 4:**
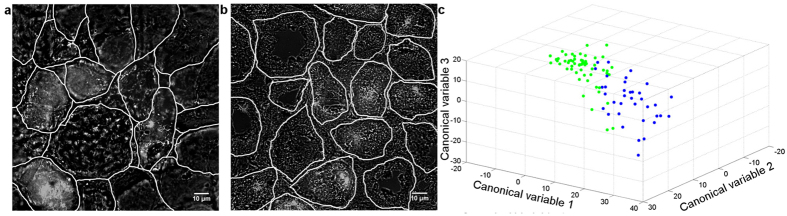
Discrimination of diabetic tissue from healthy controls using AF images. (**a,b**) the top score principal component images superimposed with a DIC image, in addition renal tubules and glomeruli have been segmented in white. (**a**) Renal tissues from a healthy control showing normal cortical architecture. (**b**) Renal tissue from a diabetic mouse showing distorted tubules. (**c**) PCA and LDA project cell feature vectors onto three new canonical variables. This space is optimised to allow successful discrimination of diabetic tissue cells (green) vs. the control cells (blue).

Diabetes is a common metabolic disorder that increases oxidative stress in the kidney. Oxidative stress due to generation of ROS has a critical role in the pathophysiology of diabetic nephropathy^44^. NADH, the major source of ROS, is recognised as key mediator of renal fibrosis^45^, commonly associated with diabetic nephropathy^46^. Here, we show that diabetic and healthy tissue can be distinguished by using suitably chosen cellular features. In this work we used streptozotocin (STZ) induced eNOS knockout mice which develop advanced diabetic nephropathy similar to human disease^47^,^48^,^49^characterised by fibrosis as well as glomerulosclerosis, tubular atrophy and tubulo interstitial inflammation^50^. Figure 4 shows an image of a freshly frozen tissue slice from control and diabetic mice. By using spectral AF we have been able to identify cell membranes without staining, thus enabling segmentation of tissue into cells and subsequent cell population analysis. A suitably chosen set of features (see Supplementary Table 1) was able to clearly distinguish between cells from diabetic mice and healthy control animals. Diabetic changes in cell metabolism can be observed spectrally because oxidative state of tissue affects the balance of spectrally different tissue fluorophores, in particular NADH and FAD^51^, while fibrotic changes are captured by texture features. We emphasise that AF features are highly informative in fresh frozen (thawed) tissue samples which are more uneven than cultures of adherent cells, and thus more challenging to image.

### Assessing developmental competence in fixed embryos

**Figure 5:**
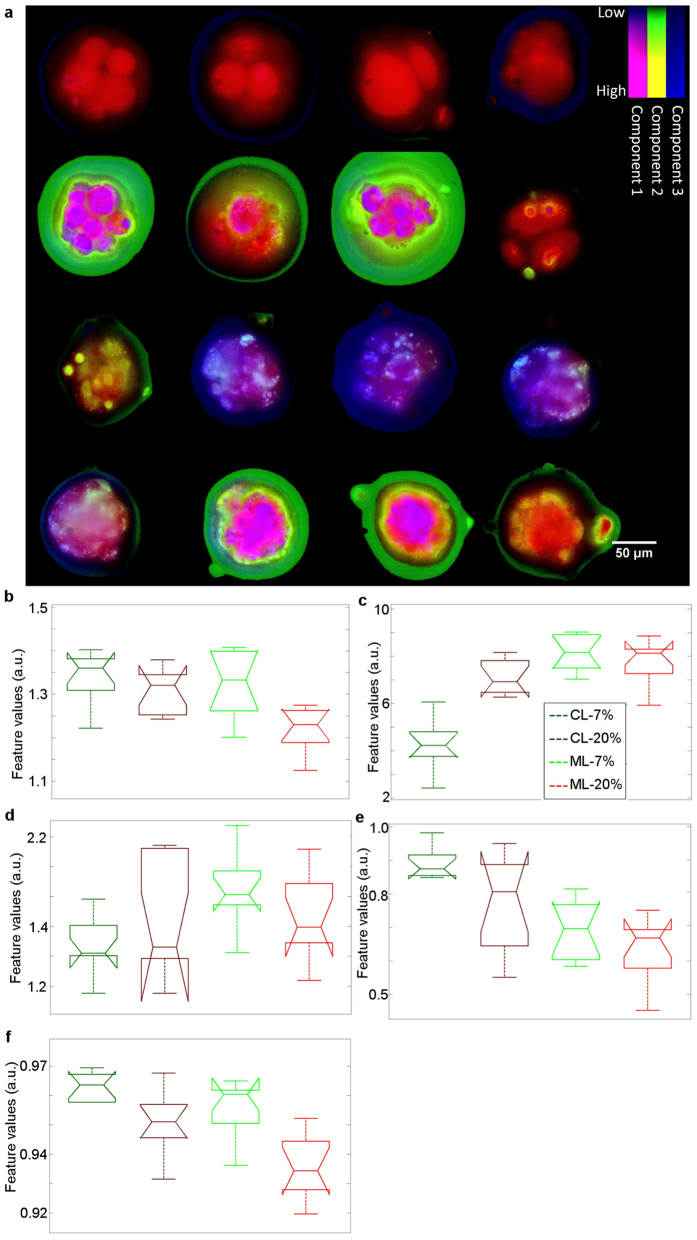
Multispectral imaging of fixed bovine pre-implantation embryos after culturing in the presence of 7% O_2_ (optimal) vs. 20% O_2_ (oxidative stress), 5 days post fertilisation. Within the oxygen culture environments, embryos were classed as developmentally delayed (cleavage stage, CL) or on-time (morula stage, ML). (**a**) Projection of pixel spectrums into a lower dimensional subspace highlights the diversity of embryo spectral content. The top row is developmentally delayed, cultured under 7% O_2_ (CL-7%), the second row is developmentally delayed, cultured under 20% O_2_ (CL-20%), and the third row is on-time cultured under 7% O_2_ (ML-7%) and the fourth row is on-time cultured under 20% O_2_ (ML-20%). The three strips in the colour key show the top three PCA components (zero values are black). The columns have no meaning. (**b–f**) Box plots for five discriminating features extracted from the data. From left to right in each panel: dark green-CL-7%; dark brown: CL-20%, light green: ML-7%, light brown: ML-20% (**b**) Band ratio (channel 3/channel 15); (**c**) Band ratio (channel 5/channel 6); (**d**) band ratio (channel 8/channel 14); (**e**) Texture in channel 4; (**f**) Texture in channel 6. We can see that the degree of heterogeneity increased in the embryos exposed to 20% O_2_.

*In vitro* produced bovine embryos from 24 h post *in vitro* fertilisation were exposed to two oxygen concentrations for 5 days of culture; 7% and 20% O_2_. These represent optimal conditions and oxidative stress respectively, see Supplementary Note 1^52^,^53^. Within each oxygen concentration, two different developmental stages of embryo were imaged on day 5 (see Fig. 5); 1) cleavage stage embryos (“arrested”, regarded as developmentally retarded, hence considered unsuitable for subsequent pregnancy establishment) and 2) morula stage (“on-time”, regarded as the stage of development embryos should be at on Day 5).Under the culture conditions described (Supplementary Note 1), morula stage embryos cultured under 7% O_2_ have a higher potential for pregnancy establishment following embryo transfer, whereas cleavage stage embryos from culture under 20% O_2_are the least likely to establish a pregnancy^52^,^53^.The obtained spectral image data were transformed using PCA in order to identify distinct spectral components within each image data set. Using the three most significant distinct spectral components obtained by PCA, we replotted the embryo images by using the intensity of red, green and blue (RGB) colours to convey the intensity of these PCA components (Fig. 5a). This allowed us to instantly visualise clear spectral changes between the different embryo types. To further quantify the differences between these embryo types we generated a suite of features based on spectral channel images using intensity, intensity ratio, correlation, texture and morphology metrics and statistical measures (see Supplementary Table 1). A selection of these is demonstrated in Fig. 5b, showing distinct and significant differences. In particular, we found different spectral properties within the embryos exposed to different O_2_ concentrations at specific developmental stages(for example 7% O_2_ morula have more homogeneous spectral profiles compared to 20% O_2_ morula).

## Conclusions

Fluorescence microscopy images of biological samples are a rich and underutilised source of quantitative information. Here, we demonstrate how to harness the information contained in spectral properties of cell AF obtained in an inexpensive system easily retrofitted on a standard epifluorescence microscope. We explain how to (a) extract and present multiple cell features in a way that highlights any chosen criterion of interest; (b) capture and deal with cell heterogeneity, subpopulations and cell discrimination; and (c) extract biologically significant cell/tissue information in a manner that is of intuitive interest to the researcher or clinician, in particular enabling verification of hypotheses. Contrary to common belief, AF provides clear distinguishing signatures of biological processes across a whole spectrum of conditions and sample types.

Our study has, for the first time, enabled the interpretation of high content AF information from cells and tissues in a way that yields new biological insights. Non-invasive screening and selection of cells is of key significance for future clinical therapies^54^. The simple, rapid and inexpensive method described here has a vast array of potential applications from enabling discoveries of unique cell subpopulations through the support of drug screening processes, to improved understanding of disease, diagnostics and therapeutics. With its ability to answer broad questions and the capacity for high throughput and automation the method presented here will contribute to improved quantification and objectivity in biological and biomedical research.

## Additional Information

**How to cite this article**: Gosnell, M. E. *et al.* Quantitative non-invasive cell characterisation and discrimination based on multispectral autofluorescence features. *Sci. Rep.* **6**, 23453; doi: 10.1038/srep23453 (2016).

## Supplementary Material

Supplementary Information
