# Supplementary material for: Quantitative non-invasive cell characterisation and discrimination based on multispectral autofluorescence features
Source: Sci Rep. 2016 Mar 31;6:23453. doi: 10.1038/srep23453 (PMC4814840; doi:10.1038/srep23453)
Supplement: Supplementary Information [file srep23453-s1.pdf]

Quantitative non-invasive cell characterisation and discrimination based on multispectral autofluorescence features

Martin E. Gosnell[1,2], Ayad G. Anwer[2], Saabah B. Mahbub[2], Sandeep Menon Pernichery[2], David W. Inglis[2], Partho P. Adhikary[3], Jalal A. Jazayeri[3], Michael A. Cahill[3], Sonia Saad[4], Carol A. Pollock[4], Melanie L. Sutton-McDowall[5,6], Jeremy G. Thompson[5,6], Ewa M. Goldys[2]

[1] Quantitative Pty Ltd ABN 17 165 684 186, www.quantitative.net.au, tel. +614 22 498 630

[2] ARC Centre of Excellence for Nanoscale Biophotonics, Macquarie University. North Ryde 2109, NSW Australia

[3] School of Biomedical Sciences, Charles Sturt University, Wagga Wagga, NSW, 2678, Australia

[4]Kolling Institute of Medical Research, Royal North Shore Hospital/Northern Clinical School, University of Sydney, Pacific Hwy, St Leonards, NSW 2065, Australia.

[5] School of Paediatrics and Reproductive Health, Robinson Research Institute, ARC Centre of Excellence in Nanoscale Biophotonics, Institute for Photonics and Advanced Sensing, The University of Adelaide

[6] Australian Research Council Centre of Excellence for Nanoscale Biophotonics& Institute for Photonics and Advanced Sensing, The University of Adelaide, North Terrace, Adelaide, South Australia, 5005, Australia

Supplementary Material

Supplementary Note 1

Preparation of biological samples

1.1. Adipose Derived Stem Cells

Stem Pro Human Adipose Derived Stem Cells (ADSCs) were obtained from Life Technologies, R7788115. These mesenchymal stem cells (MSCs) cells were isolated from human adipose tissue, collected during liposuction procedures and cryo-preserved from primary culture by Invitrogen. Prior to cryopreservation by Invitrogen, the ADSCs were expanded for one passage in MesenPRO-RS™ Medium (Life Technologies, 12746-012)[1]. Whilst this may favour the culture of ADSCs, it is possible that the cell composition of the original stromal vascular fraction may have included other cells ranging from pre-adipocytes to endothelial cells, smooth muscle cells, pericytes, fibroblasts, and blood cells. We verified the concordance of our ADCSs with the definition of MSCs by the current Tissue Stem Cell Committee of the International Society for Cellular Therapy[2]. To this aim, antigen staining of the ADSCs has been carried out. We prepared vials containing antigen stains and cells in 50uL of DPSS containing approximately 50,000 cells, as follows:

Vial	green/Brand/amount	red/Brand/amount	far red/Brand/amount
1	CD19-FITC/eB/2.5 uL<br>Catalog No:11-091-82	CD 133-PE/M/5 uL<br>Catalog No:130-098-826	CD34-Per-5.5/eB/2.5 uL<br>Catalog No:46-0349-41
2	CD45-FITC/M/5 uL<br>Catalog No:130-098-043	CD73-PE/M/5 uL<br>Catalog No:130-097-943	HLA-DR-eFluor710/eB/2.5 uL<br>Catalog No:46-9952-41
3	CD90-FITC/eB/2.5 uL<br>Catalog No:11-0909-42	CD105-PE/eB/2.5 uL<br>Catalog No:12-1057-41	CD166 /eFluor710/ eB/2.5 uL<br>Catalog No:46-1668-41

4	CD271-FITC/M/5 uL<br>Catalog No:130-098-103	CD54-PE/eB/2.5 uL<br>Catalog No:12-0549-41	CD14-Per5.5/eB/2.5 uL<br>Catalog No:45-0149-41
5	Mouse IgG1-FITC/M/5uL<br>Catalog No: 130-098-847	Mouse IgG1-PE/M/5uL<br>Catalog No:130-098-845	none

where eB stands for eBiosciences, and M stands for Miltenyi Biotech. Vials were incubated in the dark at ~4°C for 16 minutes. An equal volume of 37% paraformaldehyde was then added followed by mixing and a further 20 minutes of cold, dark incubation. Cells were then washed in DPSS and suspended in DPSS to make up 1 mL.

Antibodies to the following antigen markers were used for characterisation by flow cytometry: CD19, CD133, CD34, CD45, CD73, HLA-DR, CD90, CD105, CD166, CD271, CD54, CD14. Flow cytometry measurements were carried out by a FacsCalibur, (Becton Dickinson) and they were completed within 3 hours. An isotype negative control Mouse IgG-FITC antibody (Miltenyi 130-098-847) and Mouse IgG1-PE, (Miltenyi 130-098-845) were used to identify a baseline for the flow cytometer signal for two fluorochromes (FITC, PE) but was not available for Per-CP. The far red baseline was set based on consistent negative expression of CD34, HLADR and CD14 in control cells. These baselines were subtracted from corresponding data. The threshold between positive and negative expression was derived from the minimum between the two peaks of a histogram of the entire data set. Using this value as a hard threshold, we consider cell intensity below this as negative expression and above as positive. According to this approach the examined cells had the following surface antigen profile.

CD19- CD34- CD45- CD73+ HLA-DR- CD90+ CD105+ CD166+

This result positively verifies that the majority of the cells within our culture comply with the biomarker expression typical of for mesenchymal stem cells[2]. For confocal microscopy imaging carried out on an SPM2 (Leica) the cells were labelled with CD90, CD54, CD146, CD14 and CD166, by using the above protocol. We verified cell viability post- labelling by using a trypan blue stain. For trypan staining 0.1 mL of 0.4% solution of trypan blue was added to 1 mL of cells. A hemocytometer was loaded and examined immediately under a microscope at low magnification. The viability of cells was calculated as:

% viable cells = [1.00 – (Number of blue cells ÷ Number of total cells)] × 100

All cells showed viability > 95%.

1.2. ADSC Culturing and Expansion

Each vial of frozen cells stored at -80°C contained ~1×10^6 cells in freezing medium. Prior to each experiment, frozen cells were thawed under sterile conditions and immediately transferred to into 50 mL sterile tube containing 15 ml of prewarmed complete MesenPro RS medium (96%(v/v) MesenProBasal medium + 2% (v/v) growth supplement + 1% (v/v) Glutamine+ 1% (v/v) Antibiotic-Antimycotic).(Complete MesenPro medium cat. no: 12746-012, growth supp, 12746-018. Glutamine, 25030-081. Antibiotic - Antimycotic, 15240-062, all supplied by Life Technologies, Australia (No incubation time, centrifuged directly). After centrifugation of cells for 5 minutes at 210 RCF the supernatant was aspirated and the pelleted cells were resuspended in complete MesenPro-Rs medium and transferred into culture dishes with seeding density of 5000 cells per cm^2. Cells were incubated at 37 °C, 5% CO_2 and 90% humidity to allow cells to adhere for several hours or overnight. The medium was replaced with fresh prewarmed complete MesenProRs medium and changed every 3-4 days. In order to maintain optimum and standardised conditions for cells during

experiments, cell subculturing was performed before cells reached a 60-80% level of confluency, taking into account that the growth rate of cells is in mid logarithmic phase prior to subculturing. The viability of cells was verified by haemocytometer to be at least 90% for each subculturing. The subculturing procedure was carried out in aseptic conditions. The medium was aspirated from cells and the cells were rinsed with Dulbeco Phosphate Buffer Saline (DPBS). For cell detachment, prewarmed TrypLE, Life Technologies, cat. no.: 12604-013 (0.5ml/10 cm^2) (3.5 ml for each 75 cm culture flask) was added and incubated at 37 °C for 7-9 minutes to achieve more than 90% cell detachment. The equivalent of twice the volume used for Tryp LE of prewarmed MesenPro RS Medium was added and the cells were transferred to 15 ml conical tube, centrifuged at 210 g for 5 minutes at room temperature, followed by removal of supernatant and re-suspension of the cell pellet in a minimal volume of prewarmed medium. The viability of cells was determined using trypan blue exclusion assay as detailed in the preceding section. The seeding density of cells was approximately 5000 cells per cm^2. The medium was replaced three to four days after seeding[3].

1.3. Osteogenic differentiation experiments

The adipose-derived stem cells were encouraged to differentiate down the osteogenic pathway using Stem Pro Osteogenic medium, (Life Technologies, A1007201) containing 45 µl of (antibiotic + antimycotic solution), (Life Technologies, 15240-062) was added to 50 ml of prepared medium. In order to induce differentiation the MesenPro RS medium was removed from cultured dishes and replaced with pre-warmed complete Osteogenic medium. Cells were seeded into 75 cm^2 culture flask, at a density of 5000 cells/ $cm^{2,}$ re-feeding with culture was performed every 3-4 days over 21 days. After 21 days of cultivation, the osteogenic culture was processed for Alizarin Red S staining (Sigma Aldrich, catalogue no A3757). The medium was removed from cells followed by rinsing once with DPBS. After fixing with 4% formaldehyde solution for 30 minutes, cells were rinsed twice with distilled water followed by staining with 2% Alizarin Red S solution (pH 4.2) for 2 to 3 minutes. Cells were visualised under light microscope after rinsing three times with distilled water[4-6].

1.4. MiaPaCa-2 cell culture and generation of stable cell line

MiaPaCa-2 pancreatic cancer cells[7] were obtained from Dr. Patsy Soon, Kolling Institute of Medical Research, Sydney. Cell identity was verified as MiaPaCa-2 (ATCC CRL-1420) by the MHTP Medical Genomics Facility (Monash University, Melbourne) following the ATCC Standards Development Organization document ASN-0002 for cell line identification via short tandem repeat profiling. MiaPaCa-2 cells were maintained as described[8] in Dulbecco's Modified Eagle's medium (DMEM-high glucose, Sigma Aldrich, D5796) supplemented with 10% bovine calf serum (Sigma Aldrich, 12133C) and 1% penicillin-streptomycin (Sigma Aldrich, P4333) (complete DMEM) at 37°C in 5% CO_2 incubator.

On the day before transfection, MiaPaCa-2 ($2x10^6$) cells were seeded onto a 6-well plate. The cells were transfected at 80% confluency. Before transfection, cells were washed with Dulbecco's phosphate-buffered saline (DPBS, Sigma Aldrich, D8537) and maintained in antibiotic-free complete DMEM. The plasmid pcDNA3.1_PGRMC1-HA_S57A/S181A3[9] (4µg) and Lipofectamine 2000 (Life Technologies, 11668-019) were mixed at 1:2 ratio and incubated for 25 min at room temperature. The mixture was then added dropwise to the wells of the culture plate. After 6 hours of incubation, cells were washed with DPBS and cultured in complete DMEM for 48 hours, after which media was replaced with complete DMEM containing 50 µg/ml Hygromycin B (EMD Millipore 400052) and cultured for 2 weeks to select for stable integration events. The PGRMC1-HA_S57A/S181A monoclonal cell line was subsequently selected by limiting dilution in complete DMEM containing hygromycin as described[9].

1.5. Preparation of kidney tissue slices

Male *enos* knockout mice on a C57BL/6 background (purchased from Jackson laboratory, USA) were used for the study as this model was endorsed by the diabetic consortium to be one of the best murine models of advanced disease[10]and are known to develop advanced diabetic nephropathy similar to human disease[11-13]. Mice were housed singly in filter top cages in a pathogen free facility and had free access to standard chow and drinking water. Diabetes was induced by a low-dose streptozotocin (STZ) protocol. Mice received intraperitoneal injections of STZ (55 mg/kg daily for 5 days) at 7–8 weeks of age. Control mice received citrate buffer injections (pH 4.5). Blood glucose was tested using a glucometer (Accuchek Nano, Roche) two weeks after STZ through tail vein blood collection. Diabetes was defined by blood glucose greater than 16 mmol/L after a six-hour daytime fast. Mice with lower glucose levels were excluded from the study. Fasting blood glucose levels were measured monthly. Long acting insulin (Insulin Glargine, Sanofi Aventis, Australia) was initiated as required from 10 weeks of age and was administered thrice weekly if the blood sugar exceeded 28 mmol/L or if they had lost weight greater than 25% from baseline. The study was approved by the Royal North Shore Hospital Ethics Committee (protocol number 1101-003A). The Australian Code of Practice for the Care and Use of Animals for Scientific Purposes was followed in this study. Animals were anaesthetised using short inhalational anaesthesia with 2% isoflurane for minor procedures. Animals at 32 weeks of age were euthanised under 2% isoflurane anaesthesia using cardiac puncture terminally. Control and diabetic kidneys were embedded in OCT compound (Miles Laboratory, Ekhart, IL, USA) and rapidly frozen in liquid nitrogen. Sections (6 µm) were then prepared using a Mikrom 500 O cryostat (Mikrom, Walldorf, Germany).They have been thawed to 36 degrees immediately before imaging.The data presented in Figure 4 have been obtained from three separate tissue slices from one diabetic and three slices from one healthy control animal.

1.6.Preparation of fixed bovine embryos

Bovine cumulus oocyte complexes (COCs) were aspirated from abattoir-derived ovaries using an 18-gauge needle and 10 ml syringe. Intact COCs with compact cumulus vestments and un-granulated ooplasms were selected in undiluted follicular fluid and washed once in *in vitro* oocyte maturation (IVM) medium. Groups of 50 COCs were transferred into 500 µl of pre-equilibrated IVM medium, overlaid with paraffin oil and cultured for 23 h at 38.5°C, 6% CO_2 in humidified air. The IVM medium was VitroMat (IVF Vet Solutions, Adelaide Australia) + 4 mg/ml fatty acid free (FAF) BSA (MP Biomedicals, Solon OH USA catalogue no. 0219989980) + 0.1 IU/ml FSH (Puregon; Organon, Oss Netherland catalogue no. 460312). Mature COCs were washed once in wash medium (VitroWash; IVF Vet Solutions, + 4 mg/ml FAF BSA) and transferred into 500 µl of IVF medium (VitroFert, IVF Vet Solutions; + 4 mg/ml FAF BSA + 10 IU/ml heparin (Hospira, catalogue no. HEPA-1-5) + 25 µM penicillamine (Sigma, catalogue no. P0310000) + 12.5 µM hypotaurine (Sigma, catalogue no. 460312) + 1.25 µM epinephrine (Sigma, catalogue no. E4250), overlaid with mineral oil. Two straws of bull sperm with proven fertility were thawed, processed using a discontinuous Percoll gradient (45%:90%; GE Healthcare catalogue no. 17-0891-01) and added to IVF wells at a final concentration of 1 x 10^6 sperm/ml. Sperm and COCs were co-cultured at 38.5°C, 6% CO_2 in humidified air. After 23 h of co-culture (Day 1), presumptive zygotes were mechanically stripped of cumulus cells by repeat pipetting in wash medium, washed once in cleavage medium (VitroCleave, IVF Vet Solutions; + 4 mg/ml FAF BSA) and groups of five embryos were transferred into 20 µl drops of pre-equilibrated cleavage medium, overlaid with paraffin oil. Embryos were cultured in either 7% O_2 (optimal conditions) or 20% O_2 (oxidative stress), 6% CO_2 in nitrogen balance at 38.5 °C. On Day 5, embryo developmental stage was assessed using a dissecting microscope. Cleaved (2-8 cell staged embryos, representing arrested embryos) and morula (on-time

development) were fixed in 4% paraformaldehyde for 30 mins at 4°C,then transferred into 0.4% paraformaldehyde in PBS and stored at 4°C.

To validate the developmental outcomes of culturing embryos at different O_2 concentrations, on Day 5, a cohort of embryos were washed once in blastocyst medium (VitroBlast, IVF Vet Solutions; + 4 mg/ml FAF BSA) and groups of five embryos were transferred in 20 µl drops of pre-equilibrated blastocyst medium, overlaid with paraffin oil. Embryos were cultured in either 7% O_2 (optimal conditions) or 20% O_2, 6% CO_2 in nitrogen balance at 38.5 °C. On Day 8, embryo developmental stages were assessed to determine the proportion of embryos that reached the blastocyst stage from fertilized population. Significantly more embryos cultured in 7% O_2 reached the blastocyst stage compared to embryos cultured in 20% O_2 (7% = 43.7 ± 1.6 % vs. 20% = 32.0 ± 2.9 % blastocyst/cleaved embryos; $P < 0.05$).

1.7. Preparing cells and tissue for spectral imaging

We imaged cultured human cell lines, fixed *in-vitro*-produced cattle preimplantation embryos and freshly excised frozen tissues from diabetic mice and controls prepared as described in Online Methods and **Supplementary Note 1**. All live cell experiments were conducted using triplicate cultures of all cell types using 35 mm plastic culture dishes with 18 mm well and # 1.5 cover slip bottoms (Cell E&G, USA, Cat number GDB0004-200). These have external 200 µm grids laser etched to assist with cellular relocation for correlative experiments. Each dish is seeded with one ml of trypsinised ADSCs (5000 cells/cm^2). Cells in all groups of dishes are incubated at 37 °C, 5% CO_2 and 90% humidity and measured at ~ 37°C in otherwise ambient conditions. Unless otherwise noted, all experiments were carried out at the same cell density, in triplicates. All images have been analysed without subjective selection.

Supplementary Note 2

2.1. Multispectral microscopy measurement technique

Our method uses fluorescence of native fluorophores commonly found in cells. In our approach images of live or fixed cells and tissues are obtained in a custom-modified wide-field fluorescence Olympus IX71 microscope with fluorescent images captured by a sensitive Andor IXON 885 camera. The microscope modification consists of attaching a Prizmatix™ fibre coupled LED light source to the fluorescent illumination port usually occupied by a Hg lamp. The Prizmatix source customised by the manufacturer provides illumination at a number of selected bands of excitation wavelengths (here, centred at 334, 365, 375, 385, 395, 405, 415, 425, 435, 455, 475, 495 nm, each about 10 nm wide). The emission is measured in the range 447nm - 700 nm with the aid of suitable dichroic mirrors and long pass filters from Semrock™. During measurements a selected field of view comprising cells or tissue is consecutively imaged at each of these spectral channels. These measurements yield the fluorescence excitation spectra measured at each pixel of the examined images.

A special imaging technique was used with preimplantation embryos which have a relatively large cross section of > 50 micrometres compared to a typical value of 10-20 micrometres for plated cells. In order to image these embryos we obtained hyperspectral images focused at 5 different z positions from the central plane to the top plane of the embryo in increments of ~15 microns. For each channel we then applied a multifocus image reconstruction algorithm to produce a single image for each channel, following a method reported in Reference [14] with some modifications. First we used the average of two images to produce image segmentation. We then determined the most in-focus regions in each image using a spatial frequency metric. Then we reconstruct the embryo image using the most in-focus sections only. Furthermore we iteratively adjusted the in-focus segments to reduce the magnitude of the discontinuities between them using the spatial

frequency of the adjoining edge sections as a guide. As in the case of plated cells and tissue sections, this procedure was repeated at each of our spectral channels, yielding fluorescence excitation spectra measured at each pixel of the reconstructed images.

2.2. Spectral channels used in this work

The list of spectral channels and the respective powers at the objective is provided below.Short-term effect of light exposure during the measurement process have been established to be negligible, as the power densities and exposures (~ 1 s) used here are comparable to those in standard fluorescence microscopy.

Spectral channel number	Excitation wavelength +/- 5 nm	Emission wavelength (bandwidth), nm	Dichroic mirror, long pass, nm	Power at objective, uW
1	334	447 (60)	409	0.5
2	365	447 (60)	409	1.1
3	375	447 (60)	409	1.8
4	334	587 (35)	532	0.1
5	365	587 (35)	532	1.5
6	375	587 (35)	532	11.5
7	385	587 (35)	532	11.3
8	395	587 (35)	532	19.4
9	405	587 (35)	532	23.5
10	415	587 (35)	532	34.0
11	425	587 (35)	532	62.6
12	435	587 (35)	532	85.9
13	455	587 (35)	532	40.3
14	475	587 (35)	532	102.7
15	495	587 (35)	532	43.1
16	405	700 (long pass)	635	23.9
17	455	700 (long pass)	635	41.4
18	495	700 (long pass)	635	94.8

2.3. Image data post-processing

The data are post-processed in the following way. Here, i denotes a channel number (here i= 1,…,18, see Supplementary Material Section 2.2) and k, l are horizontal and vertical pixel indices in the images.

1. A set of "background" reference images of a culture dish with a medium for each spectral channel $B_{raw}(i,k,l)$ is taken at the beginning of each day. These images have noise removed using a wavelet filter specifically designed to remove Poisson noise produced by EMCCD camera [15]. The images are then fitted using a 2D second order polynomial fit, producing smoothed background images $B(i,k,l)$.
2. The background images are subtracted from all corresponding channel images of tested cells $T_{raw}(i,k,l)$, producing background-corrected cell images $T(i,k,l)$.
3. At the beginning of each day we also measure our calibration fluid (a mixture of 10 uM riboflavin and 10 uM NADH with 9 parts of NADH and 1 part of riboflavin). This solution is placed in an empty culture dish and its microscopy images $C_{raw}(i,k,l)$ are taken at all channels. The reference spectrum of this calibration fluid is measured by using a standard fluorimeter (Cary Eclipse) and corrected for systems response, $f(i)$,providing a relationship between the values of fluorescence signals in the spectral channels used in our microscope and the corresponding absolute values of these fluorescence signals obtained on our fluorimeter or from the literature. (The absolute, fluorimeter-independent spectra of a fluorophore are obtained after correction for systems response; this is important in order to accurately identify the fluorophores).
4. The images of the calibration fluid with the smoothed "background" image subtracted $C_{raw}(i,k,l)$ - $B(i,k,l)$ are then smoothed by using a wavelet transform, producing a smoothed corrected calibration image $C(i,k,l)$. This procedure allow us to remove spectral distortion produced by non-flat illumination of the field of view (strictly speaking different in every channel).
5. Further, the corrected cell images $T_{flat}(i,k,l)$ are produced by division, $T_{flat}(i,k,l) = T(i,k,l) / C(i,k,l)$.
6. These corrected cell images for each channel are subsequently multiplied by appropriate values of reference spectra, $f(i)$, producing fully corrected cell images $T_{cor}(i,k,l)= f(i)\ T_{flat}(i,k,l)$. This procedure makes it possible for us to compare our unmixed spectra by using the reference spectra of specified chemicals measured by various fluorimeters and also spectra of various biochemical provided in the literature.
7. Prior to applying linear unmixing, our images $T_{cor}(i,k,l)$ are further smoothed to remove the noise by a phase-preserving Poisson noise filter [15].

Supplementary Note 3

3.1. Features used in this work

In this note and in **Supplementary Table 1** we list and discuss specific features used in this paper. Note that different features were used in each biological experiment. Specific methods used in each section of this manuscript have also been indicated, as follows:

(a): Features used for distinguishing MiaPaCa-2 pancreatic cancer cells and mutants (principal component analysis, PCA in**Fig. 1a,b,c,d,e**; targeted projection pursuit, TPP in **Fig. 1 h, i**); (b) Autofluorescence features used to predict antigen levels in adipose-derived stem cells, (TPP, **Fig. 2d**); (c)Tracking early osteogenic differentiation of ADSCs (PCA, linear discriminant analysis, LDA**, Fig. 2g-i**); (d) Cell subpopulations within ADSC can be identified in a label-free manner (PCA, LDA, **Fig. 3b**); (e) Distinguishing diabetes from healthy tissue (TPP, **Fig. 4**); (f) Assessing developmental competence of fixed embryos (PCA, **Fig. 5**).Additional details regarding calculation of these features, together with their defining equations are provided below.

Supplementary Table 1

**Section of this manuscript**	Features	
(a)	1	Variance of PCA component 1
(a)	2	Skewness of PCA component 2
(a)	3	Mean cellular PCA component 3
(a)	4	Mean intensity ratio of channel 4 and channel 9
(a)	5	Texture of channel 14 intensity
(a)	6	Mean intensity ratio of channel 9 and channel16
(a)	7	Variance of the dissimilarity between cell mean spectra and endmember component 1
(a)	8	Mean intensity ratio of channel 3 and channel 4
(a)	9	Mean intensity ratio of channel 10 and channel 17
(a)	10	Mean intensity ratio of channel 4 and channel 6
(a)	11	Texture of channel 9 intensity
(a)	12	Pixel co-occurrence of endmember component 1 and endmember component 2
(b)	1	Mean cellular intensity of brightest 10% of pixels 365 nm (Ex) 447 nm(Em)
(b)	2	Mean cellular energy (texture) of 365nm(Ex), 447 (Em)
(b)	3	Skewness of texture (wavelet filter 1 ($\emptyset_1$)) 365 nm (Ex), 447 nm (Em)
(b)	4	Mean cellular texture (wavelet filter 3 (w_1)) of 385 nm (Ex), 447 nm (Em)
(b)	5	Variance of texture (wavelet filter 1 ($\emptyset_1$)) 385 nm (Ex), 447 nm (Em)
(b)	6	Skewness of texture (wavelet filter 4 (w_2)) 395 nm (Ex), 447 nm (Em)
(b)	7	Mean cellular texture (wavelet filter 2 ($\emptyset_2$)) 405 nm (Ex), 447 nm (Em)
(b)	8	Variance of texture (wavelet filter 1 ($\emptyset_1$)) 405 nm (Ex), 587 nm (Em)
(b)	9	Variance of texture (wavelet filter 4 (w_2)) 405 nm (Ex), 587 nm Em)
(b)	10	Mean cellular pixel total intensity
(b)	11	Mean cellular intensity of brightest 10% of pixels 395 nm (Ex) 587 nm (Em)
(b)	12	Kurtosis of texture (wavelet filter 1 ($\emptyset_1$)) 405 nm (Ex), 587 nm (Em)
(b)	13	Kurtosis of texture (wavelet filter 4 (w_2)) 405 nm (Ex), 587 nm (Em)
(b)	14	Mean cellular intensity of brightest 10% of pixels 425 nm (Ex), 587 nm (Em)
(b)	15	Mean of texture (wavelet filter 3 (w_1)) 425 nm (Ex), 587 nm (Em)
(b)	16	Mean cellular intensity of brightest 10% of pixels 435 nm (Ex), 587 nm (Em)

(b)	17	Mean cellular energy (texture) of 435 nm (Ex), 587 nm (Em)
(b)	18	Mean of texture (wavelet filter 3 (w_1)) 455 nm (Ex), 587 nm (Em)
(b)	19	Variance of texture (wavelet filter 4 ((w_2)) 455 nm (Ex), 587 nm(Em)
(b)	20	Skewness of texture (wavelet filter 1 ($\emptyset_1$)) 455 nm (Ex), 587 nm (Em)
(b)	21	Mean cellular intensity of brightest 10% of pixels 475 nm (Ex), 587 nm (Em)
(b)	22	Mean cellular energy (texture) of 532 nm (Ex), 587 nm (Em)
(b)	23	Mean cellular intensity endmember component 1
(b)	24	Pixel correlation of endmember component 6 and end member component 1
(b)	25	Pixel correlation of endmember component 7 and end member component 2
(b)	26	Pixel co-occurrence of endmember component 3 and end member component 2
(b)	27	Pixel correlation of endmember component 7 and end member component 6
(b)	28	Pixel co-occurrence of endmember component 6 and end member component 1
(b)	29	Variance of the dissimilarity of the mean cellular spectrum to endmember component 6
(c)	1	Mean spectral abundance ratio (endmember component 3 + endmember component 1) / (end member component 6 + endmember component 1).
(c)	2	Mean spectral abundance ratio endmember component 3/(endmember component 3 + endmember component 1).
(c)	3	Ratio of mean band intensity, 365 nm (Ex), 447 nm (Em) / 475 nm (Ex), 587 nm(Em)
(c)	4	Variance of the dissimilarity of the mean cellular spectrum to endmember component 3
(c)	5	Ratio of mean band intensity 435 nm (Ex), 587 nm (Em) / 495 nm (Ex), 587 nm (Em)
(c)	6	Pixel correlation of endmember component 6 and total endmember component 1
(c)	7	Pixel co-occurrence of endmember component 3 and total endmember component 1 + spectral residual
(c)	8	Mean local entropy of 435 nm (Ex), 587 nm (Em)
(c)	9	Pixel co-occurrence of endmember component 3 and total endmember component 1
(c)	10	Endmember image correlation: endmember component 3 and endmember component 3 + total endmember component 1.
(c)	11	(endmember component 3 + endmember component 1) / endmember component 6
(c)	12	Variance of the dissimilarity between cell mean spectra and endmember component 1

(c)	13	Mean cellular intensity of brightest 10% of pixels 365 nm (Ex), 447 nm (Em)
(c)	14	Mean cellular intensity of brightest 10% of pixels 435 nm (Ex), 447 nm (Em)
(d)	1	(endmember component 3 + endmember component 1)/(endmember component 6 + endmember component 2)
(d)	2	(endmember component 3s / (endmember component 3s + endmember component 1))
(d)	3	Mean intensity ratio (365 nm (Ex), 447 nm (Em) / 455 nm (Ex), 587 nm (Em))
(d)	4	Variance of the dissimilarity of the mean cellular spectrum to end member component 3
(d)	5	mean intensity ratio (425 nm (Ex), 587 nm (Em) / 495 nm (Ex), 587 nm (Em))
(d)	6	Pixel correlation between endmember component 6 and total endmember component 1
(d)	7	Pixel co-occurrence of endmember component 3 and endmember component 6
(d)	8	Mean local entropy of intensity 425 nm (Ex), 587 nm (Em)
(d)	9	Pixel co-occurrence of endmember component 3 and total endmember component 1
(d)	10	(endmember component 3 + endmember component 1)/(end member component 6)
(d)	11	Variance of the dissimilarity of the mean cellular spectrum to endmember component 1
(d)	12	Mean cellular intensity of brightest 10% of pixels 365 nm, (Ex), 447 nm (Em)
(d)	13	Mean cellular intensity of brightest 10% of pixels 425 nm (Ex), 587 nm (Em)
(d)	14	Pixel correlation of endmember component 3 intensity and end member component 6
(d)	15	Pixel correlation of endmember component 3 intensity and endmember component 1
(d)	16	Pixel correlation of endmember component 3 intensity and endmember component 2
(d)	17	Pixel correlation of endmember component 1 intensity and endmember component 6
(d)	18	Pixel correlation of endmember component 2 intensity and endmember component 6
(d)	19	Pixel correlation of endmember component 1 intensity and endmember component 2
(d)	20	Kurtosis of texture 532 nm Ex), 587 nm (Em)
(d)	21	Mean cellular intensity of endmember component 3
(d)	22	Mean cellular intensity of endmember component 1
(d)	23	Mean cellular intensity of endmember component 2
(d)	24	Mean intensity ratio (365 nm (Ex), 447 nm (Em) / 365 nm (Ex), 587 nm (Em))
(d)	25	Texture of endmember component 3 intensity

(e)	1	Variance of texture (wavelet filter 1 ($\emptyset_1$)) of 365 nm (Ex), 447 nm (Em)
(e)	2	Mean cellular texture 385 nm (Ex), 587 nm (Em)
(e)	3	Mean intensity ratio (385 nm (Ex), 587 nm (Em) / 405 nm (Ex), 587 nm (Em))
(e)	4	Mean intensity ratio (415 nm (Ex), 587 nm (Em) / 455 nm (Ex), 587 nm (Em))
(e)	5	Variance of texture (wavelet filter 2 ($\emptyset_2$)) of 475 nm (Ex), 587 nm (Em)
(f)	1	Mean intensity ratio (375 nm (Ex), 447 nm (Em) / 495 nm (Ex), 587 nm (Em)])
(f)	2	Mean intensity ratio (365 nm (Ex), 587 nm (Em) / 375 nm (Ex), 587 nm (Em))
(f)	3	Mean intensity ratio (395 nm (Ex), 587 nm (Em) / 470 nm (Ex), 587 nm (Em)])
(f)	4	Mean cellular energy (texture) of 334 nm (Ex), 587 nm (Em)
(f)	5	Mean cellular (homogeneity) of 375 nm (Ex), 587 nm (Em)

Endmembers 1-6 are the six spectra obtained by unsupervised unmixing of the spectral dataset whose vectors are average cellular intensities in our spectral channels. We used the method described in Reference [16] to carry out the unmixing. Endmembers are vectors whose linear combination with coefficients adding to unity spans the entire spectral dataset (assuming no noise). Spectral abundance of an endmember is an average value of that endmember in a particular cell. Simple cellular features such as mean (average), variance, correlations as well as mean channel intensity ratios are defined in a standard way. See section Definition of Features for mathematical definitions of the remaining features."Em" stands for emission, and "Ex" for excitation.

3.2. Segmentation:

In order to calculate cellular features, cells and tissues need to be segmented into individual cells. In this work have been broadly following reference[17] where prior knowledge of shape and appearance knowledge are used to improve watershed segmentation performance. We developed a multi-level iterative watershed algorithm with the ability to make informed segmentation decisions, and further optimise its own segmentation parameters with externally provided "ground truth" examples. These "ground truth" examples are provided by the expert operator through manual polygon selection of cells using a mouse. One image containing ~30 example cells has been found sufficient for good performance. These segmentation parameters to be optimised include the extent of morphological reconstruction used prior to water shedding which attempts to close small openings in cell walls and edges and reduce over segmentation. We also optimised the parameters employed in the watershed method to determine individual settlement areas (basins) in the extended maxima or the distance algorithm[18,19]. Other watershed parameters include the degree of edge detection information employed and the suppression of irrelevant maxima.

Our algorithm has the ability to self-tune and optimise its parameter sets by comparing the results of its own segmentation with the ground truth examples using image correlation of the binary segmentation masks. Parameters are constrained into ranges known to work well on a variety of candidate cell image types, and parameter values are updated through a method of gradient descent.

In subsequent stages of iterative cell segmentation, each segment is examined for its size (number of pixels) and shape (eccentricity) and these values are compared to the central tendency of the size and shape of cells obtained from the ground truth examples. Cells larger than a set number of standard deviations from the ground truth average are either rejected if too small, or re-segmented if too large, those that pass must be of similar shape to ground truth to be accepted. In this way we reject poorly segmented regions which do not well represent single cells. All algorithms developed were coded in Matlab.

3.3. Preparation of cell images for the calculation of texture features:

Images containing cells are segmented to define a perimeter around each cell's fluorescent cytoplasm. The individual segmented cell images are overlaid onto a black background and cropped forming a rectangular matrix of non-negative values. The median value of the non-zero pixels is then subtracted from all non-zero pixels, greatly reducing the sharp edge around the perimeter of the cells whilst retaining most of the obvious texture.

3.4. Definitions of features

Haralick features have been used previously for the classification of microscopic biological images and to discern between different protein structures[20,21]. Here we use these features applied directly to fluorescent images to obtain a broad description of organelle protein structure and spatial arrangement of fluorescent intracellular compartments. An effective method of obtaining a suite of textural features is by use of a co-occurrence matrix[22,23]. Here we use a computationally efficient means of calculating these features[24].

For each image we define a grey level co-occurrence matrix ,$p_{d,\theta}$. To obtain this matrix, we first divide our pixel intensities into N_g =8 gray level bins. We then focus on a selected pixel in our image and consider the (binned) intensity of the pixel adjacent to it (at a distance of one pixel ($d = 1$)),at a specific angle θ. If that adjacent pixel has the same grey intensity as the selected one the value of co-occurrence is 1. Then we add these co-occurrence values over all pixels in the entire image and divide the entire matrix by the number of such co-occurrences. Each entry in this matrix is the probability $p_{d,\theta}(i,j)$ that a pixel with a quantised grey value i is adjacent to the pixel with a grey value j. There are four directions of adjacency with angles θ =0, 45, 90 and 135 degrees. Our co-occurrence matrices are then four 8 x 8 arrays $p_{d,\theta}(\theta = 0, 45, 90 \text{ and } 135^o)$.

Further we generate Haralick features with all four co-occurrence matrices thus obtained for our image and the maximum value of a feature thus obtained was used as our final feature.

3.4.1. Texture features based on co-occurrence matrix

We used the following Haralick **texture** features based on a co-occurrence matrix:

Correlation, defined as: $\sum_{i=1}^{N_g}\sum_{j=1}^{N_g}\frac{(i-\mu_i)(j-\mu_j)p_{d,\theta}(i,j)}{\sigma_i\sigma_j}$

Here, $\mu_i, \mu_j, \sigma_i, \sigma_j$ are the means and standard deviations of partial probability density functions derived from $p_{d,\theta}(i,j)$.

Energy (also known as angular second moment) defined as: $\sum_{i=1}^{N_g}\sum_{j=1}^{N_g}p_{d,\theta}(i,j)^2$

Homogeneity (also known as inverse difference moment) defined as: $\sum_{i=1}^{N_g}\sum_{j=1}^{N_g}\frac{p_{d,\theta}(i,j)}{1+|i-j|}$

The remaining features (iv)-(ix) do not involve the co-occurrence matrix.

3.4.2. Local Entropy feature

The local entropy is based on the calculation of pair-wise entropy values of a centre pixel with its local neighbouring pixel intensities. The size of the neighbourhood chosen here is 9 pixels from the centre pixel. Thus 360 neighbouring pixel intensity values are used in the calculation of entropy at that pixel site.

To estimate local entropy, each of the i neighbour pixel intensity values is binned into one of sixteen intensity bins equally spanning the 0-255 range of grey intensity levels. From this, we construct an estimated probability distribution $P(x_i)$ for the neighbouring pixel intensity values occurring within a bin x_i.

Local entropy is then defined as: $-\sum_i P(x_i) log_2 P(x_i)$

We add that a maximum entropy or uncertainty occurs when this distribution is normal or random, therefore any local structure is likely to yield a lower entropy value.

Local entropy is defined for a specific pixel. To calculate the local entropy feature for a cell we add local entropies for each pixel in that cell.

3.4.3. Dissimilarity feature

The similarity between two n-dimensional spectra, y is quantified by using the scalar invariant spectral angle metric[25,26].

Dissimilarity is then defined as $\cos^{-1}\left(\frac{\langle x,y\rangle}{\|x\|_2\|y\|_2}\right)$

where $\langle .,.\rangle$ is a dot product and $\|.\|_2$ is Euclidean norm.

In this work we use the dissimilarity applied to the average cellular spectra.

3.4.4. Pixel correlation feature

Correlation between two intensity images is calculated using the expression for the Pearson's correlation coefficient r where images are first reshaped into two vectors, X and Y, corresponding to the two images. Each of the vectors comprises appropriately ordered intensities of their all pixels. Each vector has length n equal to the number of pixels in the image, and $\bar{X}$ is the mean of vector X.

Pixel correlation is then defined as: $r = \frac{\sum_{i=1}^{n}(X_i-\bar{X})(Y_i-\bar{Y})}{\sqrt{\sum_{i=1}^{n}(X_i-\bar{X})^2}\sqrt{\sum_{i=1}^{n}(Y_i-\bar{Y})^2}}$

3.4.5. Pixel co-occurrence

In order to calculate pixel co-occurrence for two images, both images first undergo thresholding to assign zero to noisy background pixels. The remaining pixels are then non-zero. The two images are expressed as vectors X and Y. The co-occurrence metric C is a measure of the number of corresponding pixel pair vectors X and Y being simultaneously non-zero (having a logical conjunction of 1), expressed as a percentage of the total number of non-zero pixels in vector X denoted$\tilde{x}$.

Pixel co-occurrence is then given by: $C = \frac{\widetilde{x \wedge y}}{\tilde{x}}$

3.4.6. Skewness feature

For the **skewness** we use its sample estimate given by: $\frac{\frac{1}{n}\sum_{i=1}^{n}(x_i-\bar{x})^3}{\left[\frac{1}{n-1}\sum_{i=1}^{n}(x_i-\bar{x})^2\right]^{\frac{3}{2}}}$

Here, n is the total number of pixels in the image and x_i are pixel intensity values in an image.

3.4.7. Kurtosis feature

For the **kurtosis** we use its sample estimate given by: $k = \frac{\frac{1}{n}\sum_{i=1}^{n}(x_i-\bar{x})^4}{\left[\frac{1}{n}\sum_{i=1}^{n}(x_i-\bar{x})^2\right]^2} - 3$

Here, n is the total number of pixels in the image and x_i are pixel intensity values in an image.

3.5. Wavelet Filters

Wavelets enable improved capture of spectral information from a signal compared with standard frequency spectra produced by Fourier analysis. They help to divide the signal into different scale components[24]. For example, one could use a mother wavelet which is sensitive to high frequency components and scaled versions of this mother wavelet being sensitive to low frequency components. The extension of this idea, multiwavelet filters have proved to be a computationally efficient method of capturing textural information from filters or banks of filters with attractive attributes with potentially lossless coverage of the frequency spectrum. For a detailed discussion of the filter properties, numerical function values, coefficients and application to image processing the reader is directed to the work by Strela[25], for more details on multiwavelet filters see references[26-30].

The procedure of applying a filter to an image is as follows. A filter is a vector of real numbers, $F(n)$ whose dimension is *M*, here *M*=8. The variable *P* is the number of pixels in a single row or column in an image (does not have to be the same) and *i* is the pixel index, (*i=1,…P*). Our "sampled signal" obtained in our experiment, $s(i)$(the pixel intensities of all pixels in a row or a column) is then convoluted, on a pixel-by-pixel basis with the filter.

The convolution of our discrete$F(n)$ and $s(i)$ produces the filtered signal, $s_{filtered}(i)$, according to the equation:

$$s_{filtered}(i) = \sum_{n=-M}^{M} F(n)s\,(i-n)$$

This $s_{filtered}(i)$ contains the pixel values in the filtered row or column.

In this work we use multiwavelet filter banks, which are a generalisation of single filters.

The multiwavelet filter bank of four filters $\emptyset_1, \emptyset_2, w_1, w_2,$ or, more specifically, $\emptyset_1(n), \emptyset_2(n), w_1(n), w_2\,(n) (n = 1,..8)$chosen for generating our features were based on Reference[26].

$$\emptyset_1 = \left[\frac{3}{5}, \frac{4\sqrt{2}}{5}, \frac{3}{5}, 0,0,0,0\,,0\right]$$

$$\emptyset_2 = \left[-\frac{1}{10\sqrt{2}}, -\frac{3}{10}, -\frac{9}{10\sqrt{2}}, 1, \frac{9}{10\sqrt{2}}, -\frac{3}{10}, -\frac{3}{10}, 0\right]$$

$$w_1 = \frac{1}{10}\left[\frac{1}{\sqrt{2}}, -3, \frac{9}{\sqrt{2}}, -10, \frac{9}{\sqrt{2}}, -3, \frac{-1}{\sqrt{2}}, 0\right]$$

$$w_2 = \frac{1}{10}\left[1, 3\sqrt{2}, -9, 0, 9, 3\sqrt{2}, -1, 0\right]$$

The procedure of applying our multiwavelet filter bank is as follows (see Figure below).

We first apply the filters $\emptyset_1, \emptyset_2, w_1, w_2$ to each row in the original image. This produces four images L_1, L_2, H_1, H_2. In the next stage we apply the same four filters in turn to these first transformed images, but this time we apply the filters columnwise. This produces 16 new images as shown in the figure.

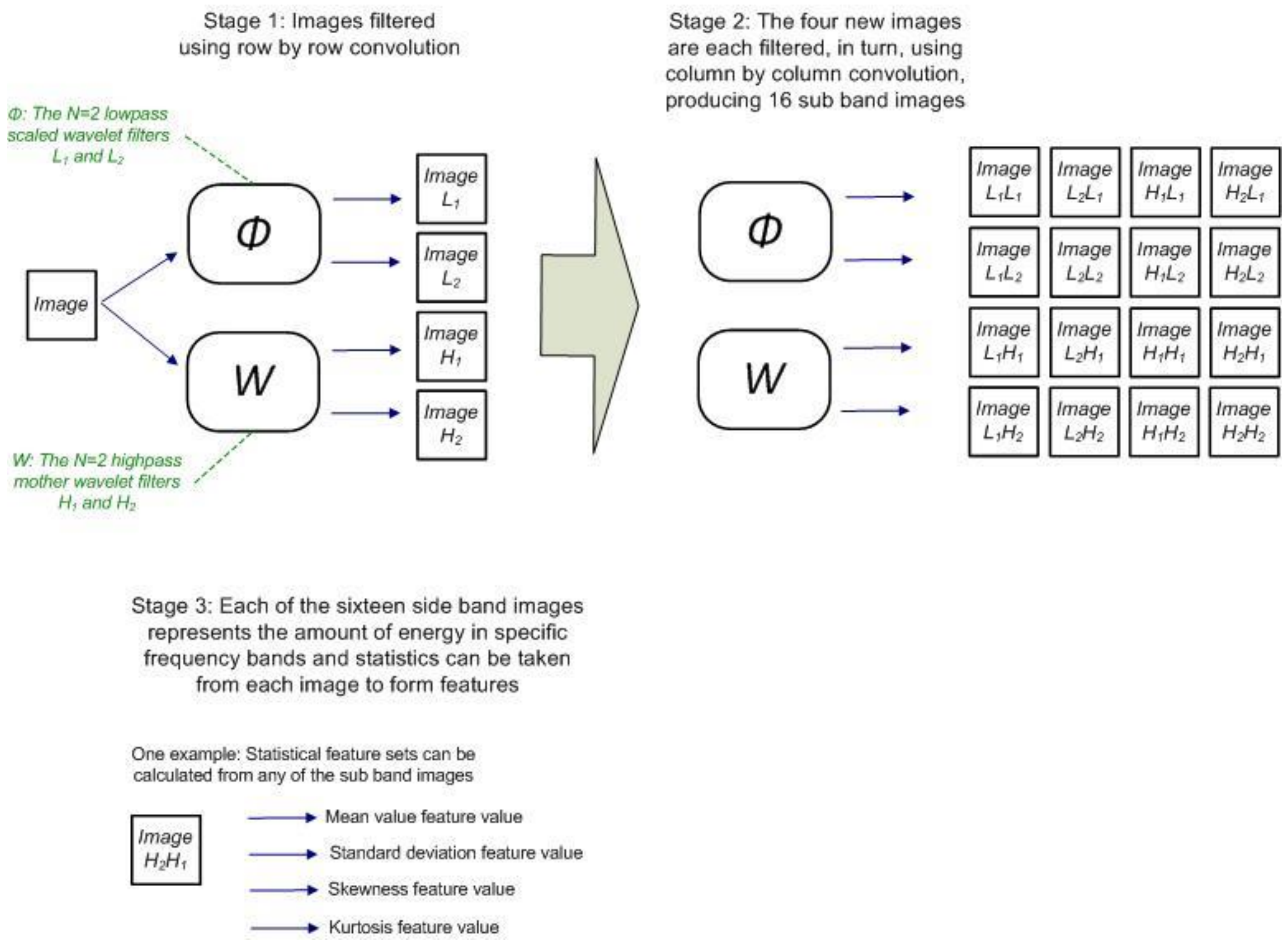

Once the filtered image is produced, we generate image features such as those listed in Supplementary Table 1.

Supplementary Note 4

4.1. Classification performance

To test the ability of this method to successfully classify cells based on spectral features a cross-validation test was employed (see schematic diagram in **Fig. 1**). The test involved partitioning the data taken from a single cell type into three sets used in each of three steps: (1) feature selection, (2) training a deterministic classifier and (3) testing. In order to make the test more stringent, we partitioned the data by using distinct cell cultures from each of triplicate culture dishes for each of the three steps. It is known that individual cultures of cells tend to develop as a community and thus any intra-culture variance may place more difficulty on classification.

The steps are described as follows.

Spectral image stacks from cells in the first culture-dish undergo a PCA transformation which allows most of the signal from all 18 spectral channels to be captured into one highly informative grey intensity image. For some data sets, particularly those with low signal to noise ratio, we use a modified PCA transform which may use a covariance model of the noise and background such that the projected image is most orthogonal to these unwanted image components, such transforms as noise adjusted PCA and maximum noise fraction are described in References[31,32]. The purpose of this first stage is to wholly represent the fluorescent cellular cytoplasm and to differentiate it from the background as well as possible. These steps produce a good quality image to which we apply our semi-supervised cell segmentation algorithm. First, an expert selects a small training set of approximately thirty cells. Using these cells as a model, the computer automatically selects other similar cells out and extracts them from the image. The outcome is manually verified. The algorithm has been described in more details in the description to **Supplementary Table 1.**

Once cells have been extracted from the images, a set of features are generated for each cell. Here, we used features listed in **Supplementary Table 1**. In principle, alternative features set (both cell-average spectral features and morphology features capturing cell image information on a pixel-by-pixel basis) could also be used, as the methodology will identify the best combinations of whatever has been taken as input. The process of feature selection is designed to provide the best distinction between two classes of cells (such as mutated cells and unmutated controls). The initial stage of feature selection employs univariate testing of individual features. Those features for which the inter class separation is highest and which meet the significance criterion are identified. The significance criterion is set at 0.05 * 1/(size of feature set) which ensures we are working to a 95% confidence level regardless of the size of the feature set from which we are selecting. From this new feature set a multivariate feature selection method is employed to select a final minimal set of distinguishing features between the two classes of cells under investigation. The number of features selected is limited to be no higher than the square root of the number of observations within the class set of smallest size. The multivariate feature selection method employs a genetic algorithm to find the best set of features. The process starts by randomly selecting a number of feature sets and calculating a fitness value for each set. The best sets are retained and also mated to produce offspring sets comprising of some of each of the features within each parent set. A few more random sets are added and the very worst performing sets are removed. Also added to the new population of feature sets are copies of the better performing sets with some random mutation, where one of the elements of the set is randomly altered. The entire process is reiterated until a stopping criterion is reached, either number of iterations or a ceiling fitness value. To calculate the fitness value, all observations for a set of features undergo a principal component analysis producing a new set of uncorrelated variables. Then a discriminatory analysis is performed which again finds a new set of basis vectors in which the principal

component data are expressed, but which provide maximum inter-class separation. Once the data are projected onto these new axes the fitness function is calculated based on the total inter-class statistical distance.

In the second step we use the data obtained from cells in the second culture dish to train a deterministic classifier. Such classifiers reduce the likelihood of overtraining, require less data than other types and tend to be more robust. The classifier uses a number of different methods to obtain a posterior probability of each cell class membership, Mahalanobis[33] quadratic or linear distance. Before the classifier is applied, the data is transformed using principal components analysis to remove unwanted correlation between variables.

The final step employing the data from cells in the third culture dish is used to test the classifier. Again, the data is transformed using principal components analysis to remove unwanted correlation between variables.

The output assigns each observation (a combination of specific cell and specific feature) a probability of being a member of each class of features. Since we have two classes of cells the overall classifier performance is then represented using a Receiver Operating Characteristic (ROC) curve[34], where a threshold probability value is used to determine the sensitivity and specificity across the range. A perfect classifier would produce an area under the ROC (AUROC) curve of one, whereas an unrelated classifier would give an AUROC of 0.5 (**Figure 1**).

4.2. Statistical hypothesis testing

As described in the manuscript, in order to statistically analyse the separation of two cell groups classified by using the above approach, we carried out an LDA projection of our data. This is a projection of the multidimensional vector space span by the features used to classify these two groups of cells onto a one-dimensional line (direction) which is optimized to obtain the highest separation of the two cell groups. This makes it possible to produce histograms of cell distributions in these two cell groups. Then standard statistical tests can be used to test the difference or identity of these histograms. In our case a non-parametric Kolmogorov-Smirnov test was used to reject the null hypothesis that the cells in both classes came from the same distributions.

Supplementary Note5

5.1. Identification of subpopulations

The method to identify cell subpopulations used here imitates human decision-making when inspecting flow cytometry data. The program checks whether the data clusters in our multidimensional feature space are sufficiently well separated. We first calculate average cell abundances of the cellular features under investigation. We then decide what is the maximum number of subpopulations we wish to find in each data group (K=2 in the presented analysis). Then for all groups within all variables, the data undergo an unsupervised and non-deterministic mixture modelling. Each produced solution is then verified and accepted only if it meets our criteria. The criteria are that the subpopulations cannot be trivial in size (here we regard 30% as a minimum), the subpopulations must have a statistical separation greater than unity. Finally the subpopulations must pass a Kolomogorov-Smirnov test of having different distributions at $p<0.05$.

Supplementary Figure 1

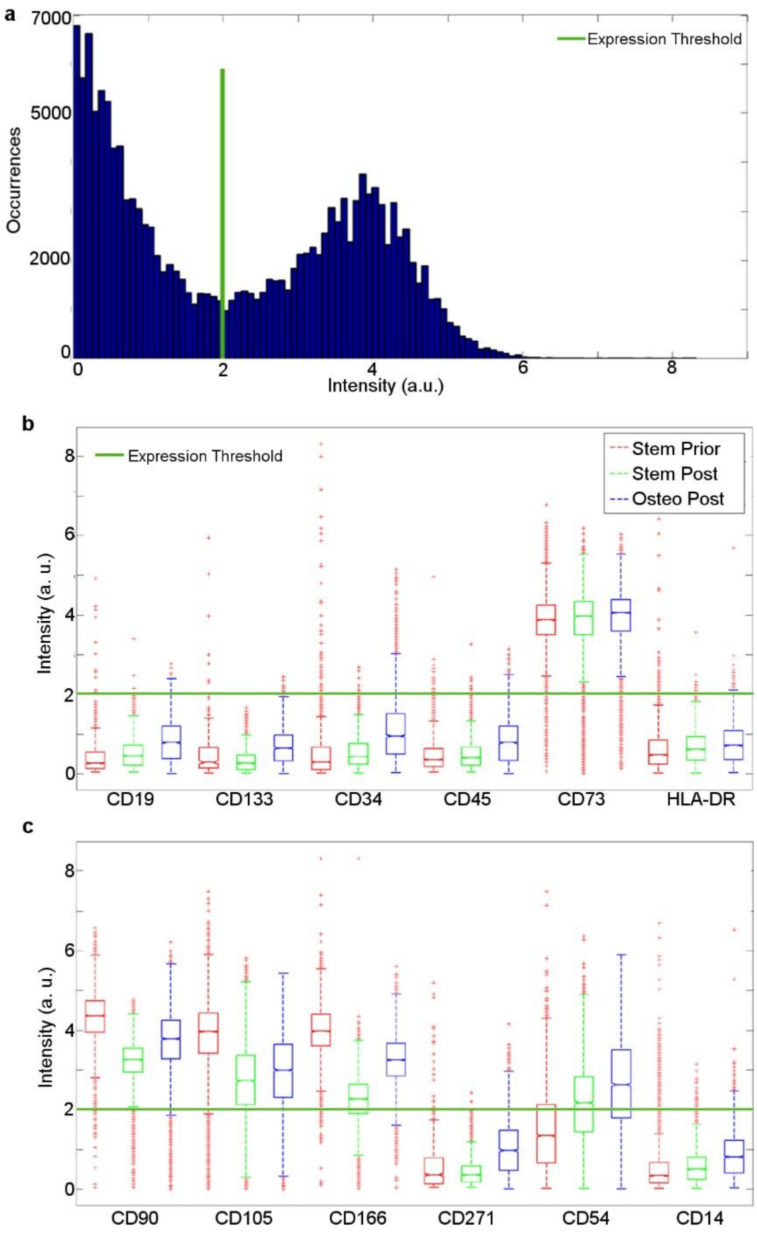

Supplementary Figure 1. Summary of flow cytometry characterisation of ADSC before and after differentiation. (a) Selection of threshold based on a histogram of aggregated flow cytometry data for all biomarkers. Green line denotes the threshold; (b, c) Cell surface antigen characterisation for ADSCs prior to experiment (day 0), ADSCs immediately after experiment (day 14) and ADSCs which have undergone osteogenic differentiation (day 14).

Supplementary Figure 2

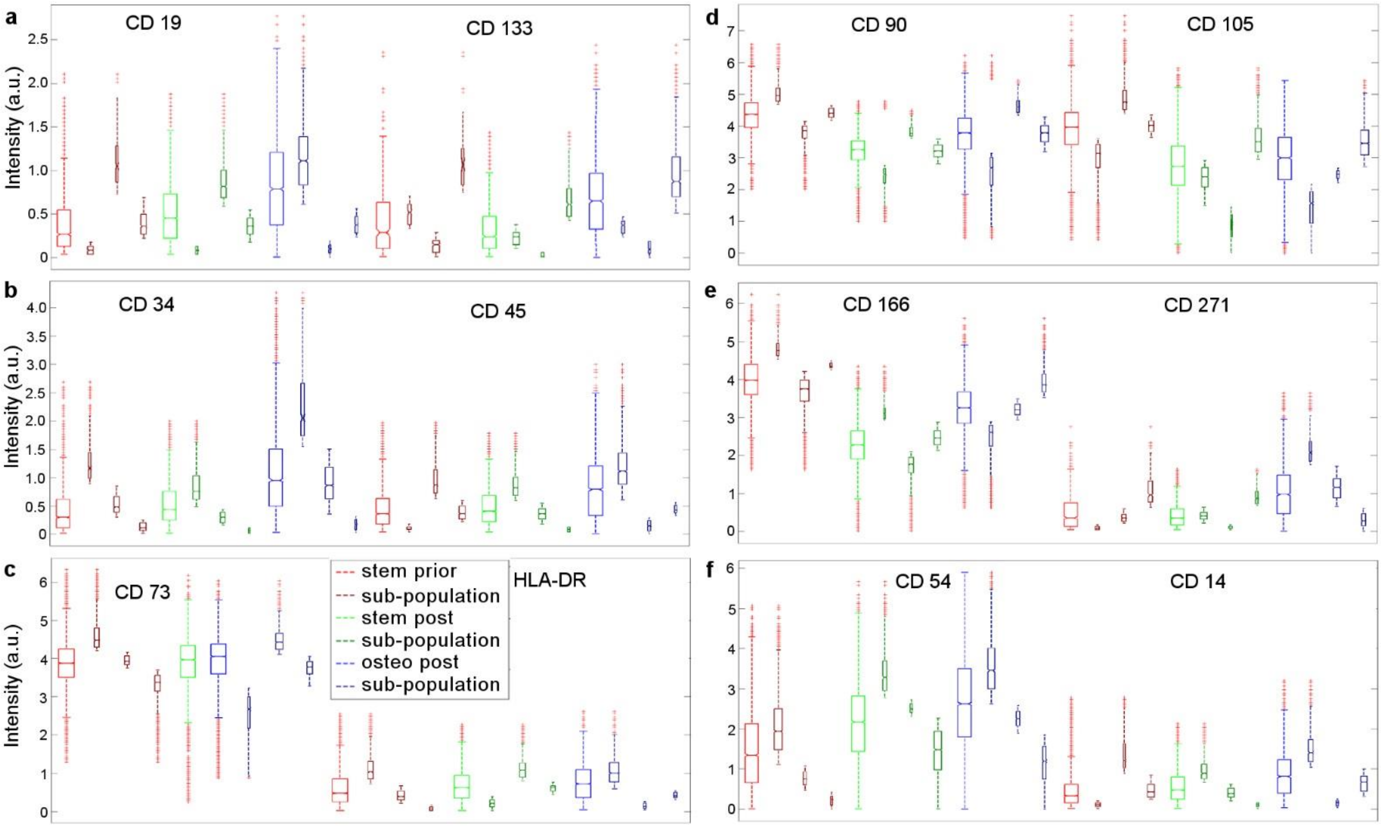

Supplementary Figure 2. Summary of subpopulation analysis in ADSCs before and after differentiation, based on surface antigen expression. Bright red: ADSCs (day 0 "stem prior"), dark red: subpopulations in ADSCs (day 0); light green: ADSCs (day 14 "stem post"), dark green: subpopulations in ADSCs (day 14 "osteo post"); light blue: differentiated ADSCs (day 14), dark blue: subpopulations in differentiated ADSCs. (a-f) – different surface biomarkers: (a) CD19 and CD133, (b) CD34, and CD45; (c) CD73and HLA-DR; (d) CD90and CD105; (e)CD166and CD271; (f) CD54 and CD14.

Supplementary Figure 3

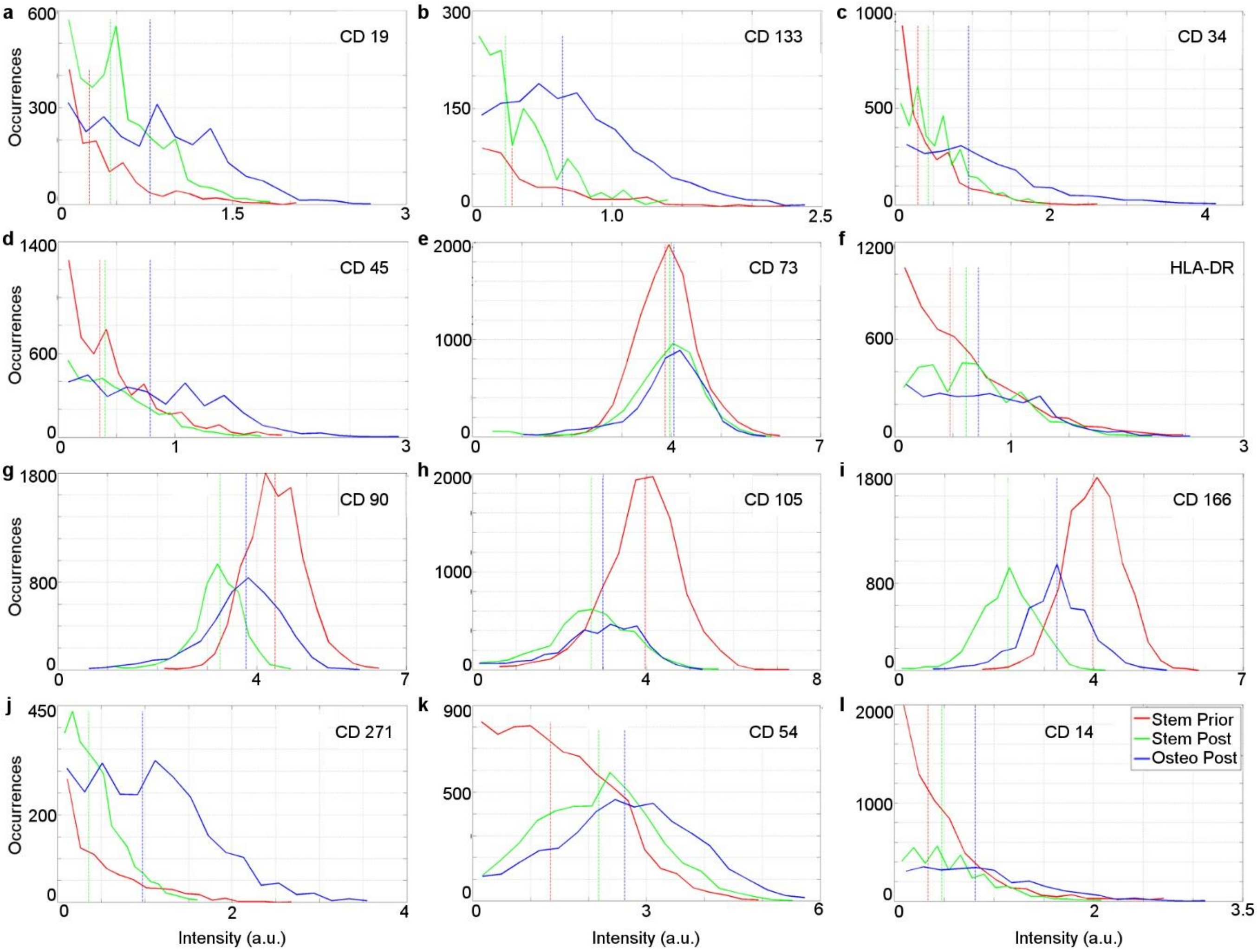

Supplementary Figure 3. Flow cytometry characterisation of the ADSCs. (a,-l) Flow cytometry signal histograms for surface antigen expression for ADSCs (day 0)- red, ADSCs (day14) – green, and differentiated ADSCs (day 14)- blue. Surface antigens are listed in the figure.

Supplementary Figure 4

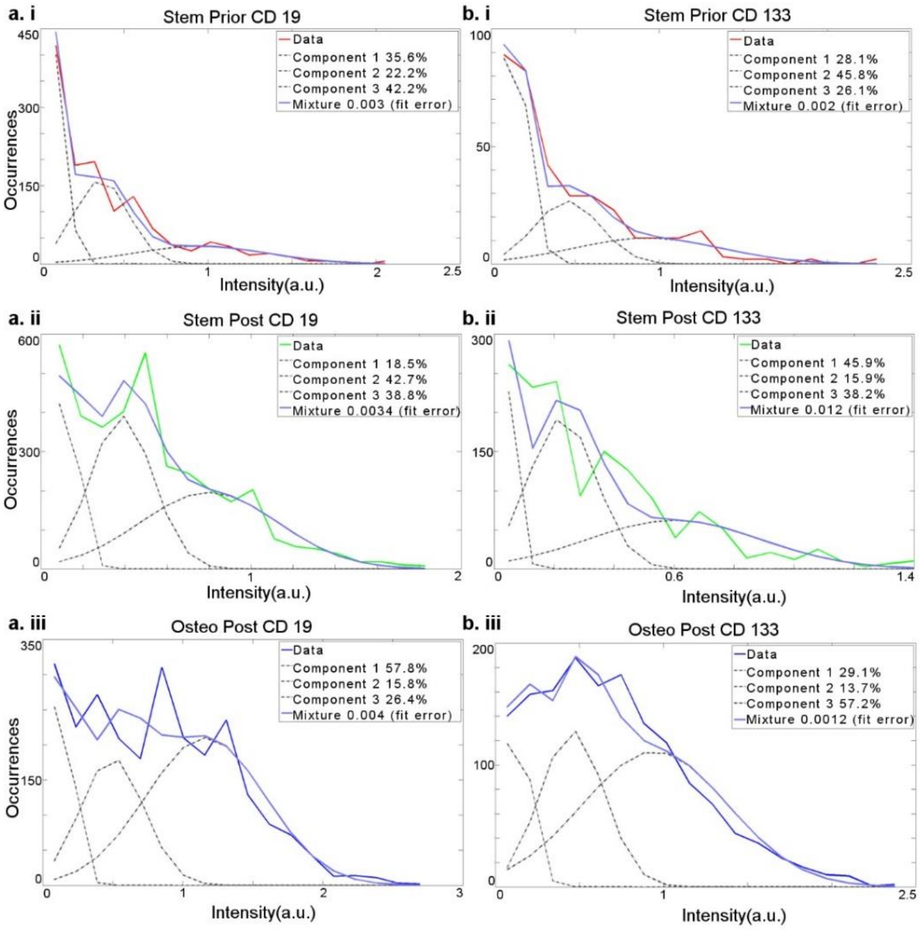

.

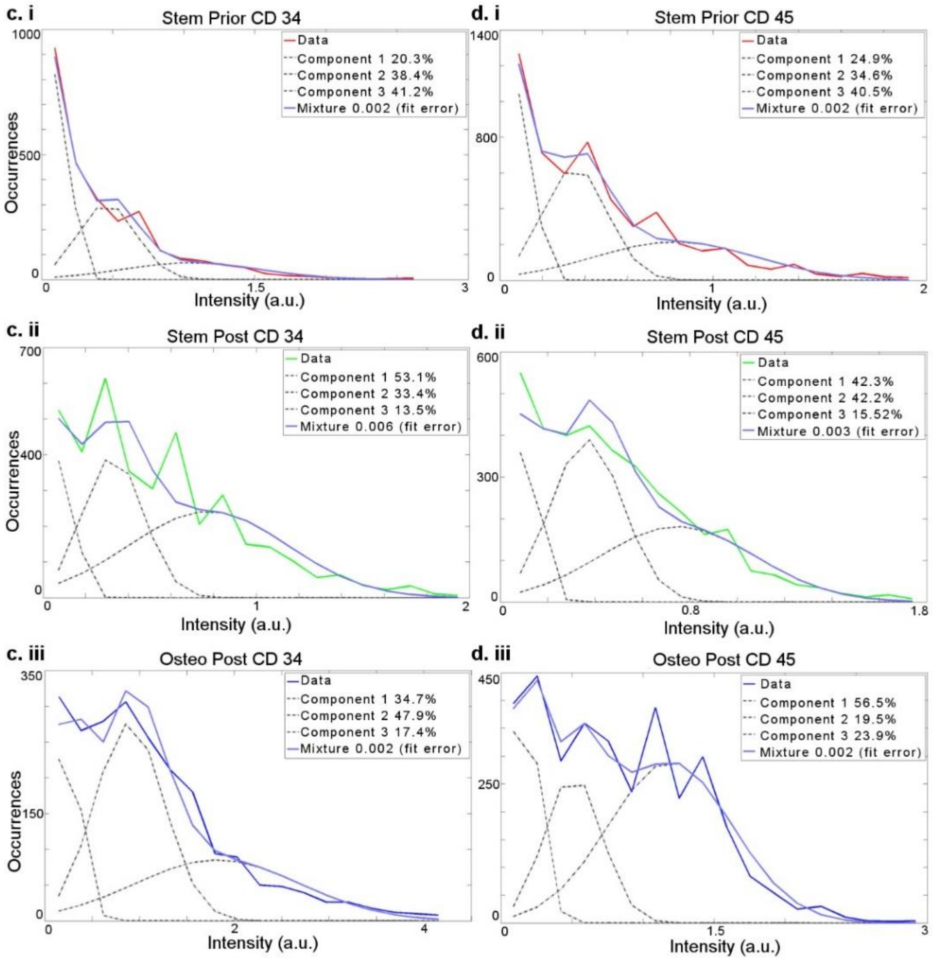
c. i
Stem Prior CD 34
Data
Component 1 20.3%
Component 2 38.4%
Component 3 41.2%
Mixture 0.002 (fit error)
Occurrences
Intensity (a.u.)
d. i
Stem Prior CD 45
Data
Component 1 24.9%
Component 2 34.6%
Component 3 40.5%
Mixture 0.002 (fit error)
Intensity (a.u.)
c. ii
Stem Post CD 34
Data
Component 1 53.1%
Component 2 33.4%
Component 3 13.5%
Mixture 0.006 (fit error)
Occurrences
Intensity (a.u.)
d. ii
Stem Post CD 45
Data
Component 1 42.3%
Component 2 42.2%
Component 3 15.52%
Mixture 0.003 (fit error)
Intensity (a.u.)
c. iii
Osteo Post CD 34
Data
Component 1 34.7%
Component 2 47.9%
Component 3 17.4%
Mixture 0.002 (fit error)
Occurrences
Intensity (a.u.)
d. iii
Osteo Post CD 45
Data
Component 1 56.5%
Component 2 19.5%
Component 3 23.9%
Mixture 0.002 (fit error)
Intensity (a.u.)

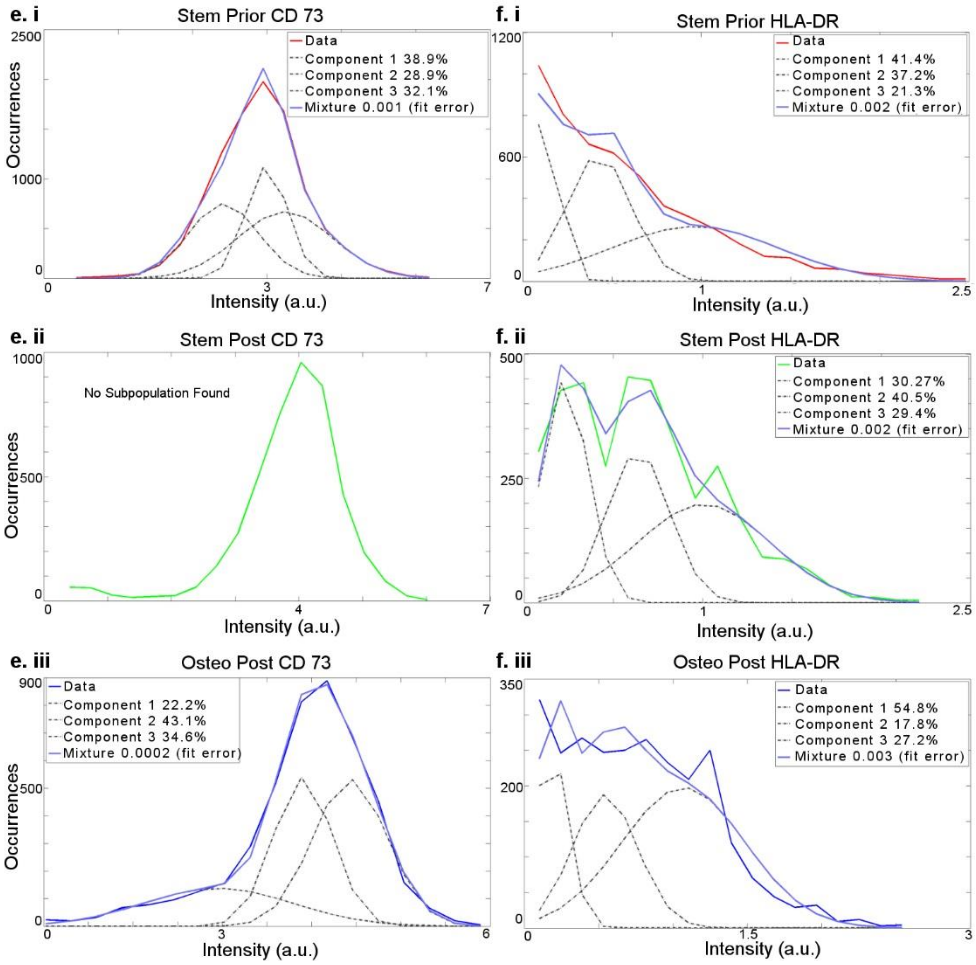

e. i
Stem Prior CD 73
Data
Component 1 38.9%
Component 2 28.9%
Component 3 32.1%
Mixture 0.001 (fit error)
Occurrences
Intensity (a.u.)
f. i
Stem Prior HLA-DR
Data
Component 1 41.4%
Component 2 37.2%
Component 3 21.3%
Mixture 0.002 (fit error)
Intensity (a.u.)
e. ii
Stem Post CD 73
No Subpopulation Found
Occurrences
Intensity (a.u.)
f. ii
Stem Post HLA-DR
Data
Component 1 30.27%
Component 2 40.5%
Component 3 29.4%
Mixture 0.002 (fit error)
Intensity (a.u.)
e. iii
Osteo Post CD 73
Data
Component 1 22.2%
Component 2 43.1%
Component 3 34.6%
Mixture 0.0002 (fit error)
Occurrences
Intensity (a.u.)
f. iii
Osteo Post HLA-DR
Data
Component 1 54.8%
Component 2 17.8%
Component 3 27.2%
Mixture 0.003 (fit error)
Intensity (a.u.)

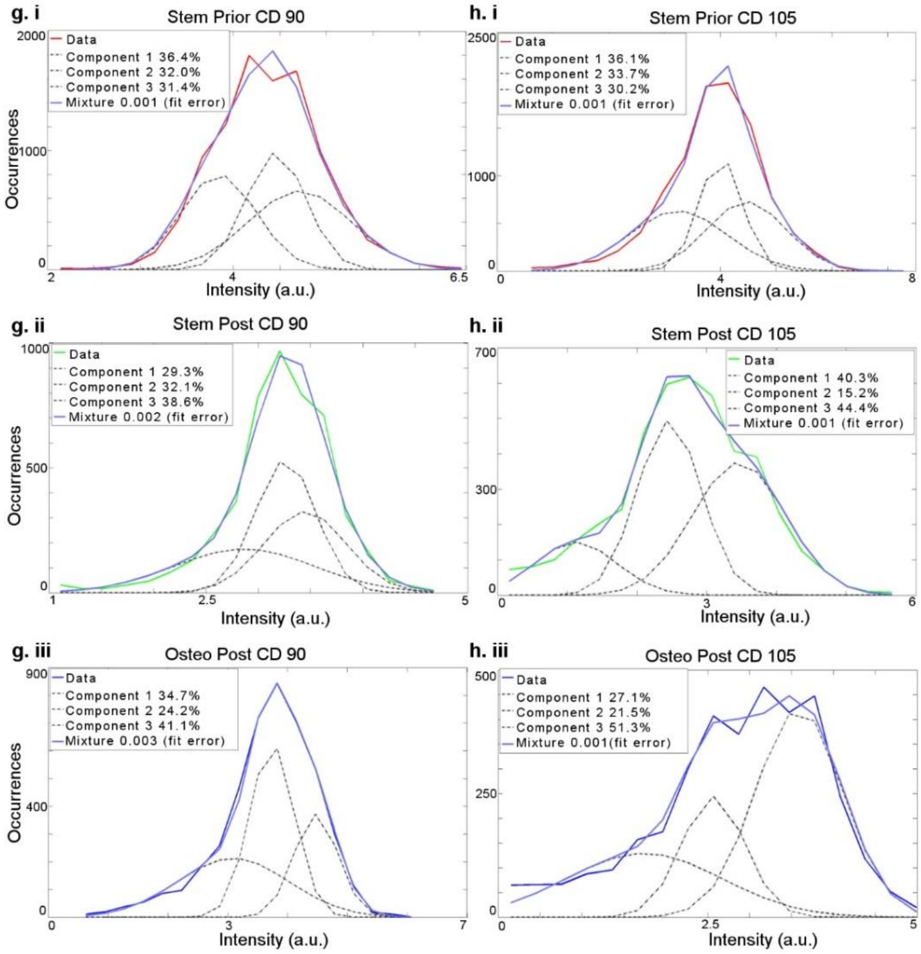
g. i
Stem Prior CD 90
Data
Component 1 36.4%
Component 2 32.0%
Component 3 31.4%
Mixture 0.001 (fit error)
Occurrences
Intensity (a.u.)
h. i
Stem Prior CD 105
Data
Component 1 36.1%
Component 2 33.7%
Component 3 30.2%
Mixture 0.001 (fit error)
Intensity (a.u.)
g. ii
Stem Post CD 90
Data
Component 1 29.3%
Component 2 32.1%
Component 3 38.6%
Mixture 0.002 (fit error)
Occurrences
Intensity (a.u.)
h. ii
Stem Post CD 105
Data
Component 1 40.3%
Component 2 15.2%
Component 3 44.4%
Mixture 0.001 (fit error)
Intensity (a.u.)
g. iii
Osteo Post CD 90
Data
Component 1 34.7%
Component 2 24.2%
Component 3 41.1%
Mixture 0.003 (fit error)
Occurrences
Intensity (a.u.)
h. iii
Osteo Post CD 105
Data
Component 1 27.1%
Component 2 21.5%
Component 3 51.3%
Mixture 0.001(fit error)
Intensity (a.u.)

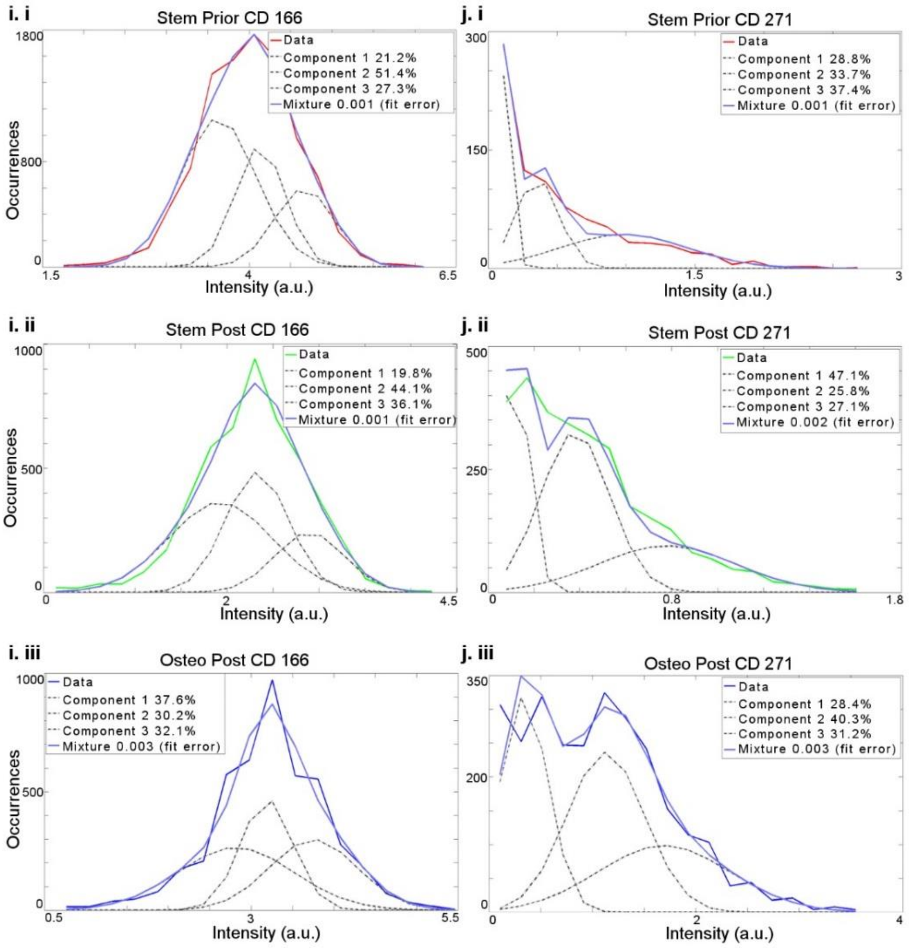
i. i
Stem Prior CD 166
Data
Component 1 21.2%
Component 2 51.4%
Component 3 27.3%
Mixture 0.001 (fit error)
Occurrences
Intensity (a.u.)
j. i
Stem Prior CD 271
Data
Component 1 28.8%
Component 2 33.7%
Component 3 37.4%
Mixture 0.001 (fit error)
Intensity (a.u.)
i. ii
Stem Post CD 166
Data
Component 1 19.8%
Component 2 44.1%
Component 3 36.1%
Mixture 0.001 (fit error)
Occurrences
Intensity (a.u.)
j. ii
Stem Post CD 271
Data
Component 1 47.1%
Component 2 25.8%
Component 3 27.1%
Mixture 0.002 (fit error)
Intensity (a.u.)
i. iii
Osteo Post CD 166
Data
Component 1 37.6%
Component 2 30.2%
Component 3 32.1%
Mixture 0.003 (fit error)
Occurrences
Intensity (a.u.)
j. iii
Osteo Post CD 271
Data
Component 1 28.4%
Component 2 40.3%
Component 3 31.2%
Mixture 0.003 (fit error)
Intensity (a.u.)

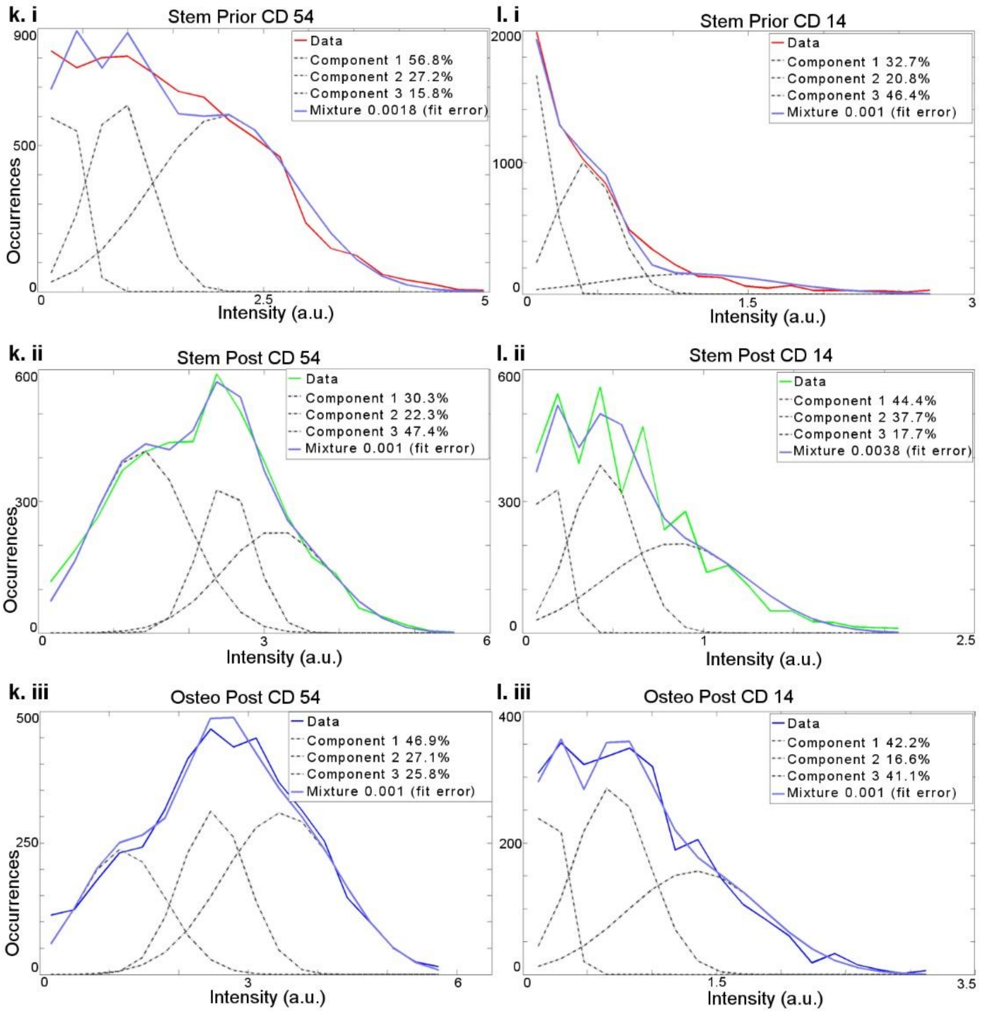

Supplementary Figure 4. Flow cytometry characterisation of the ADSCs. (a-l) Flow cytometry histograms for surface antigens as indicated in the individual panels and fits using a Gaussian mixture model with 3 Gaussian. Cells have been tested before (Stem Prior) and after (OsteoPost) osteogenic differentiation. Results indicate the existence of subpopulations with distinctive levels of specific biomarkers, particularly well visible for f,ii, and l,ii.

References:

1. Uematsu, K. et al. Tissue culture of human alveolar periosteal sheets using a stem-cell culture medium (MesenPRO-RS™): In vitro expansion of CD146-positive cells and concomitant upregulation of osteogenic potential in vivo. *Stem Cell Res* **10**, 1-19 (2013).
2. Dominici, M. et al. Minimal criteria for defining multipotent mesenchymal stromal cells. The International Society for Cellular Therapy position statement. *Cytotherapy* **8**, 315-317 (2006).
3. Hattori, H. et al. Osteogenic potential of human adipose tissue-derived stromal cells as an alternative stem cell source. *Cells Tissues Organs* **178**, 2-12 (2004).
4. Huang, J. et al. Osteoblastic differentiation of rabbit mesenchymal stem cells loaded in A carrier system of Pluronic F127 and Interpore. *Chang Gung Med J* **29**, 363 (2006).
5. Koch, T.G., Heerkens, T., Thomsen, P.D. & Betts, D.H. Isolation of mesenchymal stem cells from equine umbilical cord blood. *BMC Biotechnol* **7**, 26-29 (2007).
6. Gregory, C.A. An Alizarin red-based assay of mineralization by adherent cells in culture: comparison with cetylpyridinium chloride extraction. *Anal. Biochem* **329**, 77-84 (2004).
7. Yunis, A.A., Arimura, G.K. & Russin, D.J. Human pancreatic carcinoma (mia paca-2) in continuous culture: Sensitivity to asparaginase. *Int J Cancer* **19**, 128-135 (1977).
8. Han, E.K.-H., McGonigal, T., Butler, C., Giranda, V.L. & Luo, Y. Characterization of Akt overexpression in MiaPaCa-2 cells: prohibitin is an Akt substrate both in vitro and in cells. *Anticancer Res* **28**, 957-963 (2008).
9. Neubauer, H. et al. Breast cancer proteomics reveals correlation between estrogen receptor status and differential phosphorylation of PGRMC1. *Breast Cancer Res* **10**, R85 (2008).
10. Alpers, C.E. & Hudkins, K.L. Mouse models of diabetic nephropathy. *Curr Opin Nephrol Hypertens* **20**, 278 (2011).
11. Kanetsuna, Y. et al. Deficiency of endothelial nitric-oxide synthase confers susceptibility to diabetic nephropathy in nephropathy-resistant inbred mice. *Am J Pathol* **170**, 1473-1484 (2007).
12. Nakagawa, T. et al. Diabetic endothelial nitric oxide synthase knockout mice develop advanced diabetic nephropathy. *J Am Soc Nephrol* **18**, 539-550 (2007).
13. Zhao, H.J. et al. Endothelial nitric oxide synthase deficiency produces accelerated nephropathy in diabetic mice. *J Am Soc Nephrol* **17**, 2664-2669 (2006).
14. Li, S. & Yang, B. Multifocus image fusion using region segmentation and spatial frequency. *Image Vision Comput* **26**, 971-979 (2008).
15. Gosnell, M. E. *Unlocking the potential of spectral imaging for the characterization of cell and stem cell populations* Ph.D. thesis, Macquarie University, (2014).
16. Gosnell, M. E., Anwer, A. G., Cassano, J. C., Sue, C. M. & Goldys, E. M. Functional hyperspectral imaging captures subtle details of cell metabolism in olfactory neurosphere cells, disease-specific models of neurodegenerative disorders. *Biochim. Biophys. Acta, Mol Cell Res* **1863**, 56-63, (2016).
17. Hamarneh, G. & Li, X. Watershed segmentation using prior shape and appearance knowledge. *Image Vision Comput* **27**, 59-68 (2009).
18. Qin, Y., Wang, W., Liu, W. & Yuan, N. Extended-Maxima Transform Watershed Segmentation Algorithm for Touching Corn Kernels. *Adv. Mech. Eng* **2013** (2013).
19. Acharjya, P., Sinha, A., Sarkar, S., Dey, S. & Ghosh, S. A new Approach Of Watershed Algorithm Using Distance Transform Applied To Image Segmentation. *IJIRCCE* **1**, 185-189 (2013).
20. Chaddad, A., Tanougast, C., Dandache, A. & Bouridane, A. in Informatics and Computational Intelligence (ICI), 2011 First International Conference on 55-59 (IEEE, 2011).
21. Haralick, R.M. Statistical and structural approaches to texture. *Proceedings of the IEEE***67**, 786-804 (1979).
22. Haralick, R.M., Shanmugam, K. & Dinstein, I.H. Textural features for image classification. *Systems, Man and Cybernetics, IEEE Transactions on*, 610-621 (1973).
23. Haralick, R.M. & Shapiro, L.G. *Computer and robot vision*, Vol. 1. (Addison-Wesley Longman Publishing Co., Inc., 1992).
24. Clausi, D.A. & Zhao, Y. in Geoscience and Remote Sensing Symposium, 2002. IGARSS'02. 2002 IEEE International, Vol. 4 2453-2455 (IEEE, 2002).
25. Kruse, F. et al. The spectral image processing system (SIPS)—interactive visualization and analysis of imaging spectrometer data. *Remote Sens. Environ* **44**, 145-163 (1993).
26. Mallat, S. A wavelet tour of signal processing: the sparse way. (Academic press, 2008).
27. Strela, V., Heller, P.N., Strang, G., Topiwala, P. & Heil, C. The application of multiwavelet filterbanks to image processing. *Image Processing, IEEE Transactions on* **8**, 548-563 (1999).

28. Geronimo, J.S., Hardin, D.P. & Massopust, P.R. Fractal functions and wavelet expansions based on several scaling functions. *J. Approx. Theory* **78**, 373-401 (1994).
29. Chui, C.K. & Lian, J.-a. A study of orthonormal multi-wavelets. *Appl. Numer. Math* **20**, 273-298 (1996).
30. Tham, J.Y., Shen, L., Lee, S.L. & Tan, H.H. A general approach for analysis and application of discrete multiwavelet transforms. *Signal Processing, IEEE Transactions on* **48**, 457-464 (2000).
31. Chang, C.-I. & Du, Q. Interference and noise-adjusted principal components analysis. *Geoscience and Remote Sensing, IEEE Transactions on* **37**, 2387-2396 (1999).
32. Liu, X., Zhang, B., Gao, L. & Chen, D. A maximum noise fraction transform with improved noise estimation for hyperspectral images. *Science in China Series F: Information Sciences***52**, 1578-1587 (2009).
33. Mahalanobis, P.C. On the generalized distance in statistics. *Proc Nat Instit Sci India* **2**, 49-55 (1936).
34. Hanley, J.A. & McNeil, B.J. The meaning and use of the area under a receiver operating characteristic (ROC) curve. *Radiology* **143**, 29-36 (1982).
